# Multilevel irreversibility reveals higher-order organization of nonequilibrium interactions in human brain dynamics

**DOI:** 10.1073/pnas.2408791122

**Published:** 2025-03-07

**Authors:** Ramón Nartallo-Kaluarachchi, Leonardo Bonetti, Gemma Fernández-Rubio, Peter Vuust, Gustavo Deco, Morten L. Kringelbach, Renaud Lambiotte, Alain Goriely

**Affiliations:** ^a^Mathematical Institute, University of Oxford, Oxford OX2 6GG, United Kingdom; ^b^Centre for Eudaimonia and Human Flourishing, Linacre College, University of Oxford, Oxford OX3 9BX, United Kingdom; ^c^The Alan Turing Institute, London NW1 2DB, United Kingdom; ^d^Center for Music in the Brain, Department of Clinical Medicine, Aarhus University & The Royal Academy of Music, Aarhus 8000, Denmark; ^e^Department of Psychiatry, University of Oxford, Oxford OX3 7JX, United Kingdom; ^f^Centre for Brain and Cognition, Computational Neuroscience Group, Universitat Pompeu Fabra, Barcelona 08018, Spain; ^g^Department of Information and Communication Technologies, Universitat Pompeu Fabra, Barcelona 08018, Spain; ^h^Institució Catalana de la Recerca i Estudis Avancats, Barcelona 08010, Spain

**Keywords:** irreversibility, visibility graphs, long-term memory, higher-order interactions, neural dynamics

## Abstract

Information processing in the human brain can be modeled as a complex dynamical system operating out of equilibrium with multiple regions interacting nonlinearly. Yet, despite extensive study of the global level of nonequilibrium in the brain, quantifying the irreversibility of interactions among brain regions at multiple levels remains an unresolved challenge. Here, we present the Directed Multiplex Visibility Graph Irreversibility framework, a method for analyzing neural recordings using network analysis of time-series. Our approach constructs directed multilayer graphs from multivariate time-series where information about irreversibility can be decoded from the marginal degree distributions across the layers, which each represents a variable. This framework is able to quantify the irreversibility of every interaction in the complex system. Applying the method to magnetoencephalography recordings during a long-term memory recognition task, we quantify the multivariate irreversibility of interactions between brain regions and identify the combinations of regions which showed higher levels of nonequilibrium in their interactions. For individual regions, we find higher irreversibility in cognitive versus sensorial brain regions while for pairs, strong relationships are uncovered between cognitive and sensorial pairs in the same hemisphere. For triplets and quadruplets, the most nonequilibrium interactions are between cognitive–sensorial pairs alongside medial regions. Combining these results, we show that multilevel irreversibility offers unique insights into the higher-order, hierarchical organization of neural dynamics from the perspective of brain network dynamics.

## Significance

The brain is a complex system operating out of equilibrium with time-irreversible dynamics. Existing measures of irreversibility cannot identify which multivariate interactions display particularly nonequilibrium dynamics. In this paper, we develop a method, by constructing directed multilayer graphs from multivariate time-series, to quantify the irreversibility of each interaction in a system to identify which are markedly far from equilibrium. We analyze neural activity recorded during a long-term memory recognition task and identify key combinations of regions that are significantly irreversible, implying a strong interaction. Our results show that single processing regions, hemispheric pairs, and higher-order tuples containing hemispheric pairs alongside medial regions are the most irreversible at each level of interaction, thus illustrating this approach applied to brain network dynamics.

The human brain produces complex spatiotemporal neural dynamics across multiple time and length scales. Abstracting the brain as a large-scale network of discrete interacting regions has proved fruitful in the analysis and modeling of neural dynamics (1). Moreover, this abstraction lends neuroscientists the language and tools of statistical physics in the hope of uncovering the central mechanisms driving brain function and their links to observed neural dynamics (2, 3). For instance, recent data captured by functional imaging showed large-scale violations of detailed balance in human brain dynamics, suggesting that the brain is operating far from equilibrium (4). This fundamental observation has prompted the development of a range of techniques to provide a measure for the degree of nonequilibrium in neuroimaging time-series recorded in different conditions (5–10). These measures have shown that the degree of nonequilibrium is elevated during cognitive tasks (4–7) while reduced in both impairments of consciousness (11), sleep (10) and Alzheimer’s disease (12), indicating that nonequilibrium may be a key signature of healthy consciousness and cognition in the brain (13). Despite this, current methods are restricted to aggregate measures of nonequilibrium. We present an approach to the analysis of nonequilibrium brain dynamics that is able to measure the irreversibility of individual, higher-order interactions to gain valuable insight into the organization of neural dynamics.

The second law of thermodynamics asserts that, in the absence of entropy sinks, the average entropy of a system increases as time flows forward (14, 15). More specifically, a system at a steady-state dissipating heat to its environment causes an increase in entropy (16, 17). This results in the system breaking the detailed balance condition and results in an asymmetry in the probability of transitioning between system states (18).

This, in turn, yields macroscopically irreversible trajectories from reversible microscopic forces inducing what Eddington denoted “the arrow of time” (AoT) (19). The rate at which a system dissipates entropy, the “entropy production rate” (EPR), is a natural measure of the degree of nonequilibrium in the stationary state, as it is zero in equilibrium and positive out of equilibrium (20). Results in modern nonequilibrium thermodynamics have shown that the EPR of a nonequilibrium system can be derived from the irreversibility of observed trajectories (21–25). In particular, the EPR is given by,

[1] $$\Phi =k\lim_{\tau \to \infty }\frac{1}{\tau }D_{\text{KL}}[P\left({\left\{x\left(t\right)\right\}}_{t=0}^{\tau }\right)||P\left({\left\{x\left(\tau -t\right)\right\}}_{t=0}^{\tau }\right)],$$

where ${\left\{x\left(t\right)\right\}}_{t=0}^{\tau }$ and ${\left\{x\left(\tau -t\right)\right\}}_{t=0}^{\tau }$ represent a trajectory and its time-reversal, P(·) represents the “path probability,” the probability of observing that specific trajectory, k is Boltzmann’s constant, and $D_{\;\text{KL}}$ represents the Kullback–Leibler divergence (KLD),

[2] $$\begin{array}{cc} D_{\;\text{KL}}\left(P||Q\right) & =\int p\left(x\right)\log\left(\frac{p(x)}{q(x)}\right)\;\;dx, \end{array}$$

which measures the distance between two probability distributions P and Q with densities p and q respectively (24, 25). In the case of real-world data, trajectories are sampled at discrete time-points forming a multivariate time-series (MVTS), and the EPR is lower-bounded by the irreversibility of the observed MVTS. As a result, the irreversibility of a neural recording is a natural measure of the degree to which the neural dynamics are out of equilibrium (13).

**Fig. 1.**
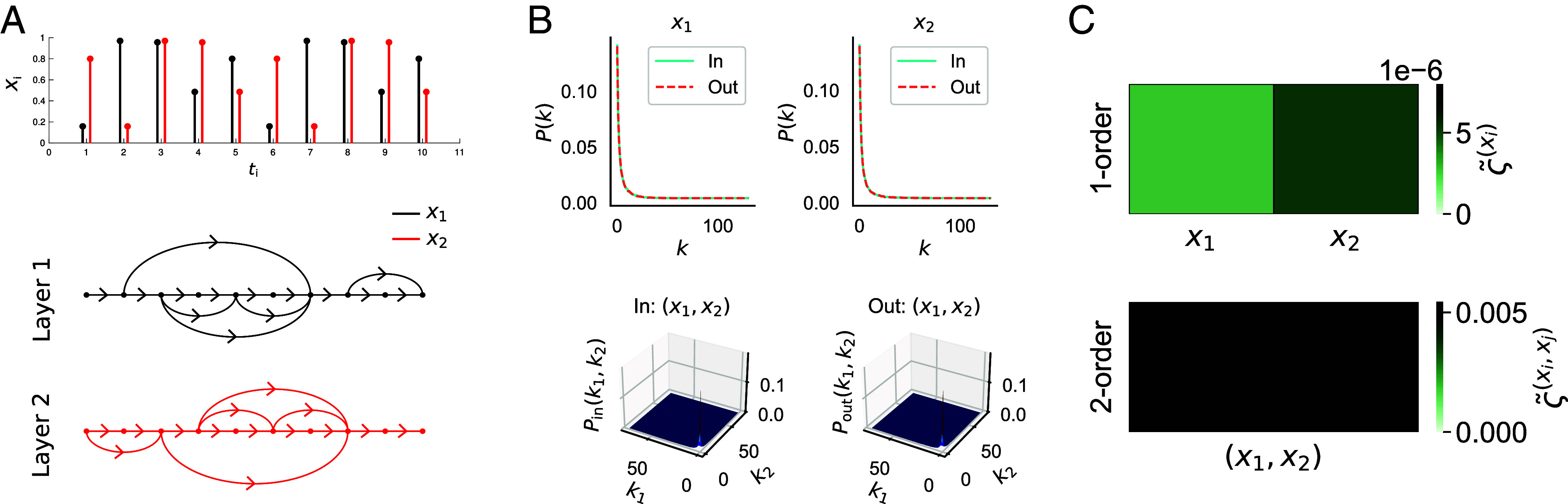
The DiMViGI workflow. The method is able to measure the irreversibility of each interaction in a multivariate time-series. It is composed of three stages, illustrated here with a random time-series of 2 variables: (*A*) First, we construct a 2-layer directed multiplex visibility graph from the multivariate time-series where each layer represents a variable and each node represents a time-point. The connections are made according to the visibility criterion defined in Eq. **7** and illustrated in Fig. 2. (*B*) Second, we calculate the in- and out-degree distributions for each tuple at each level. In the 2-variable system, there are 3 such tuples: the singletons, $\left(x_{1}\right),\left(x_{2}\right)$ and the pair $\left(x_{1},x_{2}\right)$. The *Top Left*/*Right* panels show the in- and out-degree distributions for the singletons $\left(x_{1}\right),\left(x_{2}\right)$ respectively. The *Bottom* two panels show the in- (*Left*) and out- (*Right*) degree distribution of the pair $\left(x_{1},x_{2}\right)$. (*C*) Third, we measure the Jensen–Shannon divergence of the in- and out-degree distributions for each tuple in the system. We show the 1-order irreversibility, $\varsigma^{\left(x_{1}\right)}$,$\varsigma^{\left(x_{2}\right)}$, of the singletons $\left(x_{1}\right),\left(x_{2}\right)$ (*Top*) and the 2-order irreversibility, $\varsigma^{\left(x_{1},x_{2}\right)}$, of the pair $\left(x_{1},x_{2}\right)$ (*Bottom*).

Two complimentary interpretations of the AoT in the brain have been given. First, the hierarchical organization of positions in state-space, that results from asymmetrical transition probabilities, has been linked to the dynamic hierarchical organization of brain regions (7, 26, 27). Second, the AoT has been interpreted as inducing a “causal flow” in the system where some regions emerge as information “sources” and others as “sinks” with these relationships identifiable from irreversibility analysis (7, 8). These studies for quantifying nonequilibrium in the brain approximate the global evidence for the AoT in time-series using techniques such as estimating transitions between coarse-grained states (4), with time-shifted correlations (5), machine learning (6) or with model-based approaches (7–10). However, the AoT and the corresponding production of entropy is a macroscopic property of the system, emerging from interactions between the microscopic variables at multiple scales. Recent theoretical research has shown that the AoT can be decomposed into unique contributions arising at each scale within the system (28, 29) or into spatiotemporal modes of oscillation (30), offering insights beyond a global level of nonequilibrium in the brain. Motivated by these insights, we present the Directed Multiplex Visibility Graph Irreversibility (DiMViGI) framework, as illustrated in Fig. 1, for analyzing the irreversibility of multivariate signals at multiple levels using network analysis of time-series, in particular, the visibility graph (31, 32). Using the DiMViGI framework, we investigate the irreversibility of human brain signals, captured by magnetoencephalography (MEG), during a long-term recognition task of musical sequences that utilized long-term memory (33–42). Our analysis covers all possible levels in the system and is able to capture the higher-order organization of brain regional interactions yielding interpretable insights into the neural dynamics underpinning long-term memory and auditory recognition.

## Quantifying the Arrow of Time in Multivariate Interactions

As the evidence for the AoT can be inferred from the irreversibility of observed trajectories, we focus on the quantity,

[3] $$\begin{array}{cc} \sigma & =\sum_{\Gamma }P\left(\Gamma \right)\log\frac{P(\Gamma )}{P(\Gamma^{\prime })}, \end{array}$$

where Γ is a stochastic trajectory, $\Gamma^{\prime }$ is its time-reversal and P(Γ) is the probability of observing that specific trajectory. Eq. **3** is precisely the KLD between the forward and backward path probabilities, which is a natural measure of the irreversibility of a stochastic process (23). Inspired by previous decompositions (28, 29), we note that individual interactions can have differential levels of irreversibility within a globally nonequilibrium system. Our framework aims to compute the irreversibility of individual k-tuples of variables in a MVTS in order to compare interactions at each level, defined by k. First, we consider the projection of an N−dimensional trajectory, $\Gamma ={\left\{x_{1}\left(t\right),\ldots ,x_{N}\left(t\right)\right\}}_{t=0}^{T}$, into the portion of state-space defined by the k-tuple of variables $\left(x_{i_{1}},\ldots ,x_{i_{k}}\right)$, to be the k-dimensional trajectory,

[4] $$\Gamma^{\left(x_{i_{1}},\ldots ,x_{i_{k}}\right)}={\left\{x_{i_{1}}\left(t\right),\ldots ,x_{i_{k}}\left(t\right)\right\}}_{t=0}^{T}.$$

The DiMViGI framework then quantifies the marginal irreversibility of a given tuple by approximating,

[5] $$\varsigma^{\left(x_{i_{1}},\ldots ,x_{i_{k}}\right)}=\sum_{\Gamma^{\left(x_{i_{1}},\ldots ,x_{i_{k}}\right)}}P\left(\Gamma^{\left(x_{i_{1}},\ldots ,x_{i_{k}}\right)}\right)\log\frac{P(\Gamma^{\left(x_{i_{1}},\ldots ,x_{i_{k}}\right)})}{P(\Gamma^{\prime (x_{i_{1}},\ldots ,x_{i_{k}})})},$$

using visibility graphs, as will be detailed subsequently. As a result, we are able to identify tuples of variables whose multivariate trajectory is highly irreversible indicating a strongly nonequilibrium interaction between the variables in this tuple, which also suggests the presence of a hierarchical structure within the tuple (7).

## Measuring Irreversibility with the Multiplex Visibility Graph

We build on the growing paradigm of network analysis of time-series that has gained traction in the analysis of neural signals (43, 44). These methods are characterized by mapping a time-series into a corresponding network. For instance, the visibility algorithm maps a univariate time-series into a so-called “visibility graph” (VG) (31). VGs and their variations are a powerful model-free tool for mapping a continuous-valued time-series into a discrete object. Their versatility, as well as their lack of assumptions on the underlying dynamics, has lent them to diverse applications, in particular in neuroscience (43, 44), as well as in the calculation of information-theoretic quantities from complex and chaotic dynamics (45). Explicitly, given a time-series ${\left\{X_{i}\right\}}_{i\in I}$ with time indices ${\left\{t_{i}\right\}}_{i\in I}$, where $X_{i}\in R$ and I is the index set, the VG has one node for each i∈I. Nodes i,j∈I are connected by an edge if the corresponding data-points $\left(t_{i},X_{i}\right)$ and $\left(t_{j},X_{j}\right)$ are “mutually visible” i.e. that they satisfy that, for any intermediate data-point $\left(t_{k},X_{k}\right)$ with $t_{i}<t_{k}<t_{j}$,

[6] $$X_{k}<X_{j}+\left(X_{i}-X_{j}\right)\frac{t_{j}-t_{k}}{t_{j}-t_{i}}.$$

**Fig. 2.**
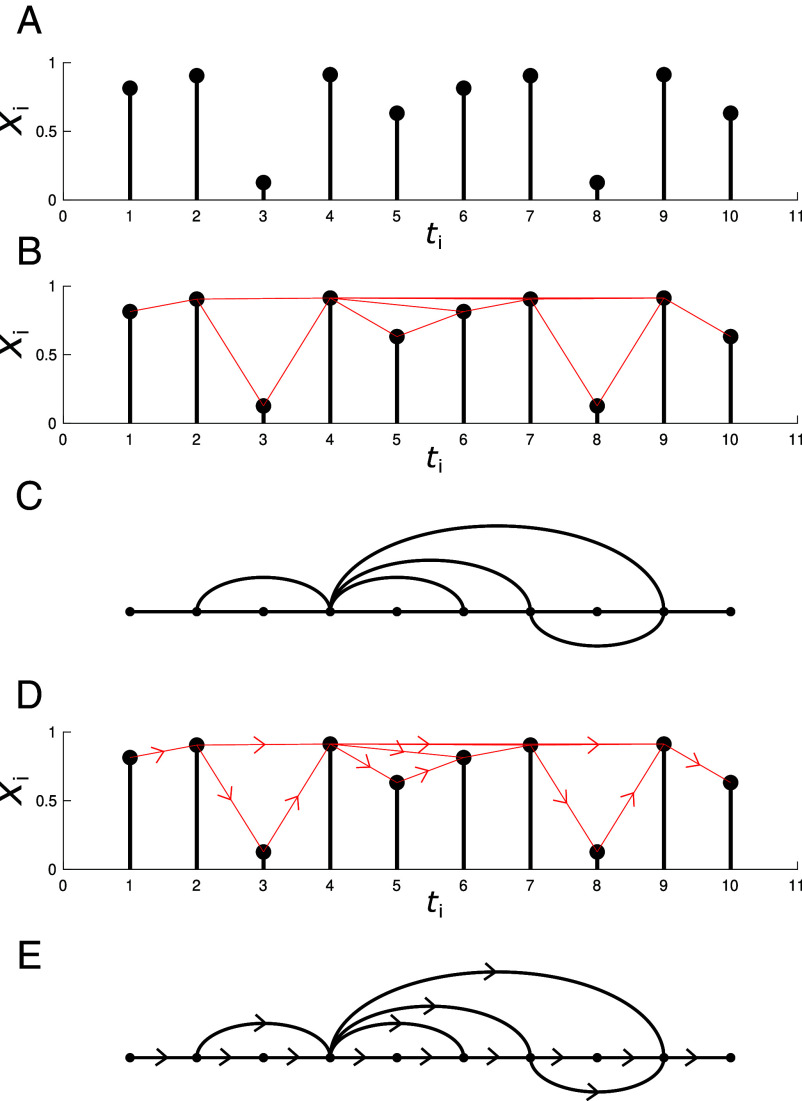
Visibility graphs. An example of a visibility and a directed visibility graph constructed from a random time-series. (*A*) A random equi-spaced time-series. (*B*) The red lines connect data points that are mutually visible. (*C*) The visibility graph associated with the random series. (*D*) A time-series showing visibility directed forward in time. (*E*) The directed visibility graph corresponding to the above series.

In geometric terms, this condition is met if $\left(t_{i},X_{i}\right)$ is visible from $\left(t_{j},X_{j}\right)$. That is, the line connecting $\left(t_{i},X_{i}\right)$ and $\left(t_{j},X_{j}\right)$ does not cross any intermediate data-points as shown in Panel (*B*) of Fig. 2. Trivially, each node is connected to its neighbors while large positive fluctuations become hubs with many connections due to their greater visibility. This construction can be naturally extended to a MVTS by considering the “multiplex visibility graph” (MVG) (46). Given a MVTS with N variables, the MVG is a multilayer graph, a so-called “multiplex,” with N independent layers with the same node base. Applying the visibility algorithm to each variable in turn yields a series of VGs which each define one layer of the MVG.

We can further generalize the VG to measure irreversibility in univariate time-series by extending the undirected VG to a time-directed counterpart (DVG) (32, 47). To do so, we simply direct the edges “forward in time.” For example, an edge connecting time-points $t_{i}<t_{j}$ is now directed i→j (see Panels *D* and *E*) of Fig. 2). We then decompose the degree d of a node into the sum of the in-going and out-going degree,

[7] $$\begin{array}{cc} d & =d_{\text{in}}+d_{\text{out}}. \end{array}$$

A univariate stationary process, X(t), is time-reversible if the trajectory $\left\{X\left(t_{1}\right),\ldots ,X\left(t_{T}\right)\right\}$ is as probable as $\left\{X\left(t_{T}\right),\ldots ,X\left(t_{1}\right)\right\}$ (48). Therefore, in the case of a reversible process, the in- and out-going degree distributions of the associated DVG should converge (32, 47). It follows that the level of irreversibility can be captured by measuring the divergence between the in- and out-going degree distributions. We extend this method to the case of MVTS. We direct the edges of the MVG such that they go forward in time yielding a directed MVG (DMVG). Since this is a multiplex graph, we can calculate the multivariate joint, over all layers, in- and out-going degree distributions, and all associated marginals.

Explicitly, we consider a MVTS with N variables and T time points, given by $\left\{X\left(t_{1}\right),\ldots ,X\left(t_{T}\right)\right\}$, where $X\left(t_{i}\right)=\left(x_{1}\left(t_{i}\right),\ldots ,x_{N}\left(t_{i}\right)\right)\in R^{N}$ and construct its associated DMVG. For a given k-tuple of variables, $\left(n_{1},\ldots ,n_{k}\right)$, we calculate the multivariate marginal in-going and out-going degree distributions:

[8] $$P_{\;\text{in}}^{\left(n_{1},\ldots ,n_{k}\right)}\left(d_{1},\ldots ,d_{k}\right),\;P_{\;\text{out}}^{\left(n_{1},\ldots ,n_{k}\right)}\left(d_{1},\ldots ,d_{k}\right),$$

where $P^{\left(n_{1},\ldots ,n_{k}\right)}\left(d_{1},\ldots ,d_{k}\right)$ is the probability of a node having degree $d_{i}$ in layer $n_{i}$ for all i simultaneously. We then compute the divergence between these particular in- and out-going marginal distributions using Jensen–Shannon divergence (JSD) (*Materials and Methods*) to obtain a measure of the k-order irreversibility,

[9] $$\begin{array}{cc} \varsigma^{\left(n_{1},\ldots ,n_{k}\right)} & =\;\text{JSD}(P_{\;\text{in}}^{\left(n_{1},\ldots ,n_{k}\right)}||P_{\;\text{out}}^{\left(n_{1},\ldots ,n_{k}\right)}). \end{array}$$

As we are considering the multivariate joint distribution, we are quantifying irreversibility in the multivariate state-space. Repeating this for all possible k-tuples in the system, we quantify the relative irreversibility of each interaction at a given level. We can repeat this process for all values of k, thus measuring irreversibility at all levels.

In summary, the DiMViGI framework, shown in Fig. 1, begins with a MVTS of neural activity. The series is mapped into the associated DMVG using the visibility algorithm. We calculate the joint in and out-degree distributions and all the possible marginal in- and out-degree distributions. We measure the JSD between the pairs of in- and out-marginals for each tuple in the system to quantify the irreversibility of that interaction. At each level k, we can then compare the relative irreversibility of each k-order interaction to identify the dominant irreversible interactions.

## Analysis of MEG During Long-Term Recognition

**Fig. 3.**
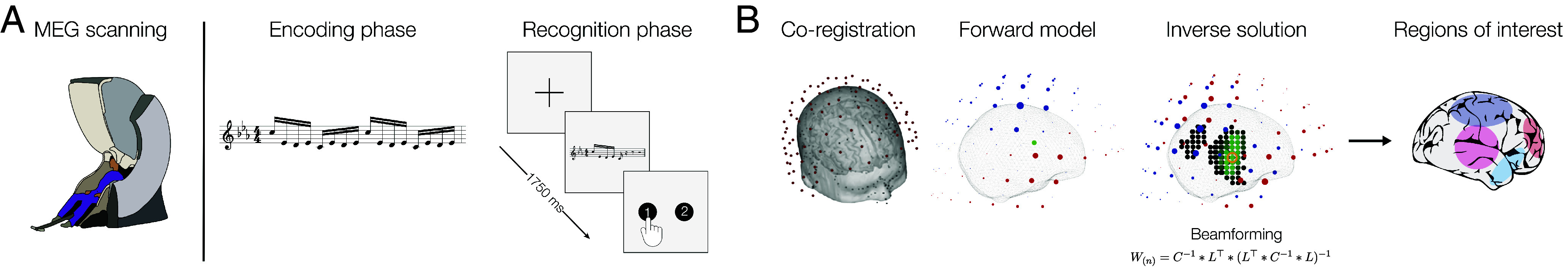
Experimental paradigm for the collection and processing of MEG data. (*A*) The brain activity in 51 participants was collected using magnetoencephalography (MEG) while they performed a long-term auditory recognition task. Participants memorized a 5 tone musical sequence. They were then played 5 further sequences of tones that were either the original sequence or a modified version. They then were requested to state whether the sequence belonged to the original music or was a varied version of the original sequences. In this analysis, we only consider the experimental condition where participants were played the original memorized sequence. (*B*) The MEG data were coregistered with the individual anatomical MRI data, and source reconstructed using a beamforming algorithm. This procedure returned one time-series for each of the 3,559 reconstructed brain sources. Six main functional brain regions (ROIs) were derived. The neural activity for each ROI was extracted yielding a multivariate time-series. For further details on the experimental set-up, see *Materials and Methods* and *SI Appendix*. For a comparison between experimental conditions, see ref. 33.

**Fig. 4.**
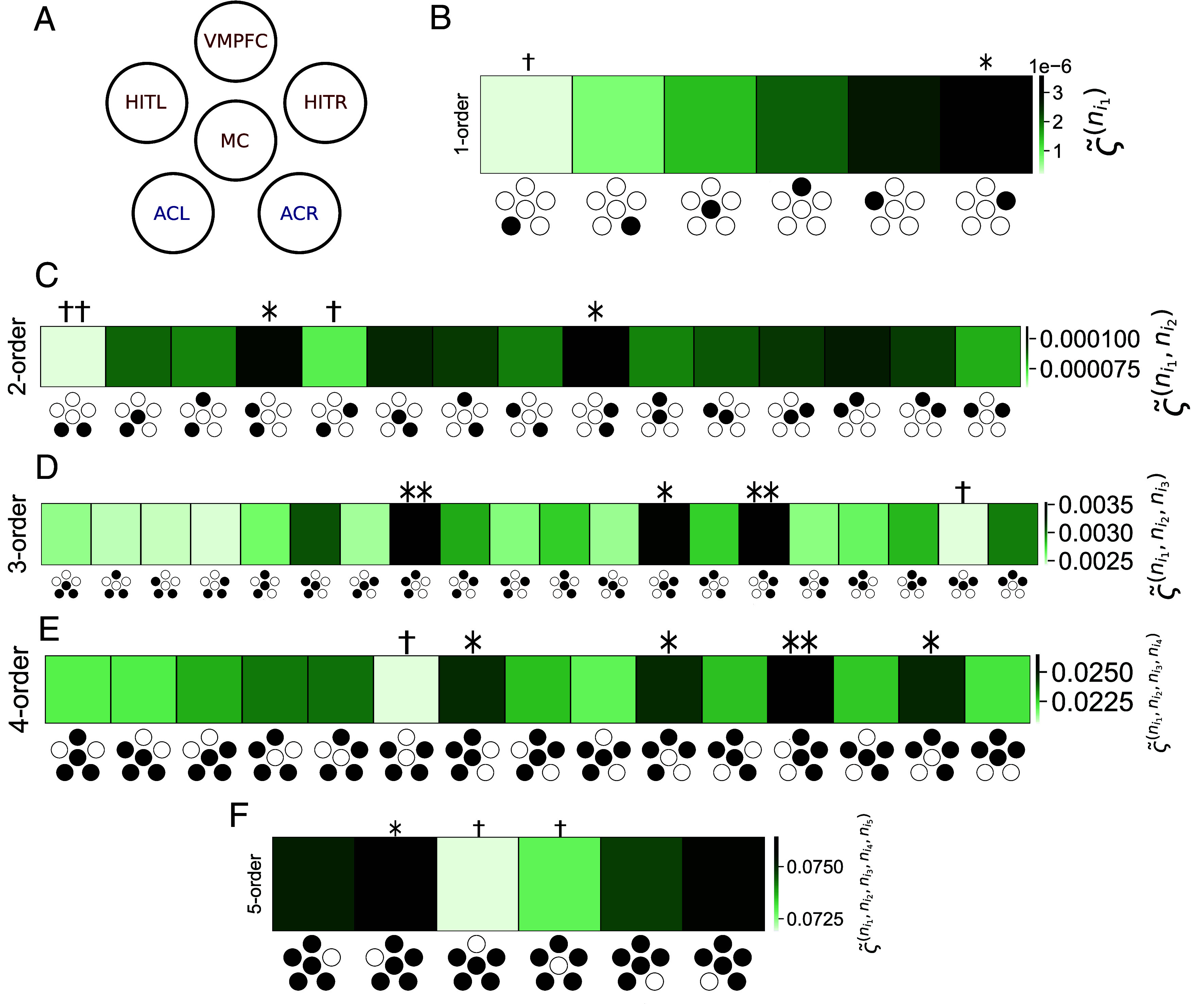
DiMViGI analysis of 6-ROI MEG recordings during a long-term memory task. The number of (∗)/(†) represents the number of SDs above/below the mean for a particular tuple at that level. (*A*) Schematic diagram showing the organization of the ROIs in the MEG recordings. The ROIs are ACL/R: auditory cortex Left/Right; MC: medial cingulate gyrus; VMPFC: ventro-medial prefrontal cortex; HITL/R: hippocampal inferior temporal cortex Left/Right. Cognitive regions are in red and sensory regions in blue. (*B*) 1-order irreversibility at cohort-level. At this level, we consider irreversibility of each signal in isolation. The hippocampal regions are the most irreversible while the sensory regions are the most reversible. (*C*) 2-order irreversibility at cohort-level. The pairs that show the most irreversibility are those that include a sensory and hippocampal pair in the same hemisphere (ACL/R, HITL/R). The most reversible pair is (ACL, ACR) which is made up of two sensory regions. (*D*) 3-order irreversibility at cohort-level. The triplets that are most irreversible are those that include an intrahemispheric sensory and hippocampal pair as well as the prefrontal cortex (ACL/R, HITL/R, VMPFC). The most reversible contains both hippocampal regions and the medial cingulate gyrus, (HITL, HITR, MC). (*E*) 4-order irreversibility at cohort-level. The quadruplets that are most irreversible are those that include a hippocampal and sensory pair and both medial regions (ACL/R, HITL/R, MC, VMPFC) and those that include both hippocampal regions, a sensory region, and the VMPFC. The most reversible is the quadruplet that contains no medial regions. (*F*) 5-order irreversibility at cohort-level. The most reversible quintuplets are those that omit a medial region, in particular, the quintuplet that omits the VMPFC.

We consider MEG recordings from 51 participants with 15 trials per participant source-localized into 6 regions of interest (ROIs) collected according to the experimental paradigm presented in Fig. 3, described in *Materials and Methods*, *SI Appendix*, and in ref. 33. The ROIs include the auditory cortices in the left and right hemispheres (ACL, ACR); the hippocampal and inferior temporal cortices in the left and right hemispheres (HITL, HITR) and two medial regions, the bilateral medial cingulate gyrus (MC) and the bilateral ventro-medial prefrontal cortex (VMPFC). Panel (*A*) of Fig. 4 shows a schematic representation of the regions. The participants performed an auditory recognition task during the MEG recordings (Fig. 3*A*). First, they memorized a short musical piece. Next, they were presented musical sequences and were requested to state whether the sequence belonged to the original music or was a varied version of the original sequences. Since differences between experimental conditions have been described in detail by Bonetti et al (33) and are beyond the scope of this work, here, we consider only one experimental condition, where participants recognized the original, previously memorized sequences.

For each participant and trial, we construct the DMVG. Next we estimate every marginal in- and out-degree distribution using each DMVG as a sample and calculate the JSD. We denote the JSD between k-dimensional degree distributions as the k-order irreversibility. Alternatively, for each participant in isolation, the degree distributions can be calculated using only their associated trials to get an estimate of the k−order irreversibility for each participant and each tuple (*SI Appendix*). However, due to the higher number of samples, the cohort-level analysis is more robust and hence is our focus in this report. The results of the DiMViGI analysis are presented in Fig. 4. We note that the darker colors represent tuples with greater irreversibility while the lighter colors reflect more reversible interactions. The icon along the x-axis indicates which tuple is being considered, with reference to the schematic in Panel (*A*) of Fig. 4, with the included regions colored in black. Furthermore, we highlight statistically significant tuples at each level. The number of (∗)/(†) indicates the number of SDs above/below the k−level mean.

We begin our analysis at 1-order. While individual (microscopic) variables are often reversible in a nonequilibrium complex system, the ROIs considered here reflect a very coarse parcellation of the brain. At this level, we are considering each ROI, which is composed of many truly microscopic variables, in isolation and note that each one shows significant irreversibility. It is clear from Panel (*B*) of Fig. 4, that the ROIs have a clear disparity in their levels of irreversibility. The sensory ROIs are more reversible than the medial and hippocampal ROIs. Furthermore, there is a skew toward the right hemisphere being more irreversible than the left. This result emerges consistently across all levels. Next, we consider the irreversibility of pairwise interactions (k=2). Panel (*C*) of Fig. 4 shows the 2-order irreversibility for all pairs. We are able to identify strongly irreversible pairs such as the intrahemispheric pairs (ACL, HITL) and (ACR, HITR). On the other hand, cross-hemispheric pairs, e.g. (ACL, ACR), are the most reversible, indicating a lack of interaction between them. The strong hemispheric symmetry in the results validates the findings, as it is an expected and intuitive observation. Panel (*D*) of Fig. 4 shows the irreversibility for each triplet interaction in the system. The highly irreversible triplets are those that include a hemispheric pair alongside a medial region, with those containing the VMPFC, a region known to drive brain dynamics during task (49), being particularly irreversible. Panel (*E*) of Fig. 4 shows that the most irreversible quadruplet interactions are composed of a hemispheric pair alongside both medial regions as well as those that contain (VMPFC, HITL, HITR) alongside a sensory region. Conversely, the quadruplet containing no medial regions, is the most reversible, and therefore has the least interaction. This is particularly interesting as this quadruplet is made up of the two most irreversible pairs yet they do not appear to interact as a foursome. Therefore, this framework is truly capturing higher-order interactions that cannot simply be decomposed into a sum of independent interactions of lower order. Finally, Panel (*F*) of Fig. 4 shows that quintuplets that contain both medial ROIs are the most irreversible. Furthermore, the quintuplet that does not contain the VMPFC has the most reversible interaction. While we have attempted to interpret the results from the perspective of the hierarchical and higher-order organization of the auditory system, we note that outliers would be expected to arise naturally due to statistical variation. Nevertheless, due to the consistency of our results across levels, for example the hemispheric symmetry that is observed at each level, such results cannot be explained purely by chance. Furthermore, a subsampling analysis shows that the error in irreversibility measurements is typically smaller than differences between tuples implying a range of statistically significant differences (*SI Appendix*).

We can interpret this result in the context of predictive coding and its links to sensory tasks (50–52), as well as through the hierarchical organization of the auditory system. The participants are exposed to a memorized tonal sequence that does not deviate from their expectation of what they were about to hear. Under the theory of predictive coding, this would result in an adjustment of a participant’s prior expectations, facilitated by asymmetric, hierarchical interactions between brain regions at multiple levels, in order to reinforce the prior expectations in light of the new sensory information (53). This in turn would lead to a cascade of interactions between key ensembles of regions whose function is optimized for the process of auditory recognition. As irreversible brain dynamics stem from irreciprocal and hierarchical interactions, such a mechanism results in marked irreversibility in the emergent dynamics (7).

## Discussion

In this study, we describe a framework for measuring the emergence of nonequilibrium dynamics, through multivariate irreversibility, at multiple system levels. We are able to capture the irreversibility of each possible interaction in a MVTS of signals. Applying the DiMViGI framework to neural recordings obtained during a long-term memory recognition task, we investigate the higher-order organization, and the associated nonequilibrium interactions, of brain regions and how they break time-reversal symmetry during an auditory recognition task. The results clearly show a broad distribution of irreversibility at each system level; hence we are able identify which interactions are particularly irreversible, which we interpret as a correlate of a hierarchical and synergistic interaction. Furthermore, we link irreversibility to hierarchical predictive coding and theorize that nonequilibrium interactions could emerge as a consequence of the modulation of prior expectations in light of new sensory information (53). According to the theory of predictive coding, this might be realized through hierarchically asymmetric interactions that, in turn, induce the emergence of irreversibility at multiple system levels (7, 54, 55). Within this context, the DiMViGI framework confirms the hierarchical organization of the auditory system (56–59), with reciprocal connections, such as those found within the auditory cortex, resulting in more reversible dynamics, and hierarchical relationships, such as those found between the auditory cortex and the hippocampus, resulting in markedly irreversible dynamics. Furthermore, our approach goes beyond typical approaches to the auditory system, such as the analysis of coactivation and functional connectivity (60, 61) or the identification of cortical-gradient hierarchies (33, 58), by uncovering higher-order interactions within the auditory system between triplets and quadruplets of brain regions. In particular, at higher orders, irreversibility reveals synergistic interactions between hippocampal, cingulate gyrus and sensory regions for the distributed processing required for audition and long-term recognition. As a result, our approach yields insights that offer a perspective on the flow of information during audition. While a recent analysis of these neural recordings with standard methods was able to identify a hierarchy of information processing in the brain during long-term recognition (33), the introduction of the DiMViGI framework appears crucial to uncovering the higher-order and nonequilibrium nature of the interactions. Such insights are opaque to traditional analyses but emerge from the unique lens of nonequilibrium statistical physics.

The implications of the framework and the associated results are multifold. First, we go beyond aggregate (4–7, 9, 10) or univariate (32, 47) measures of irreversibility, expanding the existing quiver of techniques for studying nonequilibrium in the brain to include a multilevel approach. Our technique is able to capture differences in irreversibility across scales in continuous time-series, inspired by recent theoretical work for binary variables (28, 29), that is nonspecific and can be applied to MVTS from any domain to identify particular highly nonequilibrium interactions. Our approach differs from refs. 28 and 29 as we do not attempt to measure the unique contribution to the AoT of a specific k-body interaction by discounting the irreversibility of all subinteractions contained within the tuple. Instead, we measure the irreversibility of the tuple as a whole. In *SI Appendix*, section 6, we consider an extension of our approach to relate our framework more closely to the approach of refs. 28 and 29, by measuring the unique contribution of each k−body interaction, defined recursively as,

[10] $$\begin{array}{cc} \eta^{\left(x_{i_{1}},\ldots ,x_{i_{k}}\right)} & =\varsigma^{\left(x_{i_{1}},\ldots ,x_{i_{k}}\right)}-\sum_{\Omega \subset \{x_{i_{1}},\ldots ,x_{i_{k}}\}}\eta^{\Omega }. \end{array}$$

However, we note that the exact decomposition of the EPR presented in refs. 28 and 29 relates to discrete, Markovian and multipartite dynamics and thus does not apply directly to continuous MVTS. Moreover, in *SI Appendix*, section 5 we show that irreversibility in our method only decomposes in the case of independent variables.

Our framework builds on the sustained interest in identifying higher-order interactions in neural recordings and other MVTS (62–66), particularly in information theoretic analyses of brain data that reveal how higher-order functional interactions shape neural dynamics (67–69). Notably, many higher-order frameworks are either computationally, or by formulation, restricted to studying either triplet (63, 64, 66, 67) or system-wide interactions (62), while our results extend easily to all possible levels in the system. Our framework attempts to bridge the broader discussion on higher-order mechanisms and behaviors in complex systems (70–72) with techniques from nonequilibrium thermodynamics (20) through the quantification and interpretation of multilevel irreversibility. Finally, our work further solidifies the visibility algorithm, and network analysis of time-series, as an empirically useful tool in the analysis of neural data (43, 73). The code used to implement the DiMViGI framework is available at ref. 74. The MEG data used in the study is available at ref. 75 with the preprocessing scripts available at ref. 76.

Despite these promising results, we note some nuanced limitations in our framework. While the visibility algorithm and the degree distribution approach reduces the dimension of the data, we are still computing an entropy between high-dimensional distributions which is computationally restrictive. This can be circumvented by limiting the support of the degree-distribution to exponentially improve computational efficiency while minimally affecting numerical accuracy (*SI Appendix*). Nevertheless, analyzing all possible interactions yields a combinatorial explosion, hence we opt for a coarse, low-dimensional, parcellation of the brain that allows us to analyze the system at all possible levels. However, the highlighting of individual tuples is most meaningful when there is a strong intuition about the nature of the interaction, which can be only be expected in low-dimensional parcellations where ROIs are clear, functionally segregated brain areas. Additionally, we note that our measure is undirected within the tuple, meaning we cannot identify the direction of information flow as one can with classical measures of causality (77, 78) or some approaches to the AoT (7, 8). However, we note that the AoT represents directed flow between states and not variables, meaning it is not a direct measure of causality, but instead capturing a distinct, but related, phenomena in interacting dynamics. Finally, measuring the irreversibility of finite-length time-series naturally induces a bias due to the finite sampling of the state-space (4, 29). In order to validate that the measured irreversibility emerges from nonequilibrium dynamics and not from finite-data errors, we employed both surrogate-testing using shuffled time-series and subsampling approaches to validate the significance of our results (*SI Appendix*, section 4).

A key advantage of the DiMViGI framework is the ability to scale between levels with a consistent approach. Strictly local measures such as auto- and cross-correlations are limited to individual and pairwise interactions (79, 80). On the other hand, simply applying global measures to each subset of variables in the time-series, such as coarse-graining or using a model-based measure, yields an inconsistent approach where different tuples cannot be compared fairly. Our framework extends consistently to all levels thus yielding directly comparable quantities at each level.

## Conclusions

In this work, we have introduced the Directed Multiplex Visibility Graph Irreversibility framework for measuring the irreversibility of multivariate interactions at all levels within a system. We applied this method to neural recordings during a long-term auditory recognition task to study the relative irreversibility of different interactions between brain regions. Doing so, we were able to demonstrate the hierarchical, higher-order organization of brain dynamics during tasks. This analysis suggests that reinforcement of prior expectations during an auditory recognition task is facilitated through a hierarchy of irreversible higher-order interactions in the brain, an observation that we link to both the mechanisms of predictive coding and the hierarchical structure of the auditory system. Furthermore, we highlighted the particular combinations of cognitive and sensorial regions that are preferentially recruited during audition and long-term recognition. This framework is nonspecific and provides a general tool for investigating higher-order interactions and nonequilibrium dynamics in MVTS emerging from other complex systems.

## Materials and Methods

### Estimating Degree Distributions from Finite Samples.

For each sample, a MVTS, we construct the DMVG, defined by the multiplex adjacency matrix, A,

[11] $$A_{\mathrm{ij}}^{\left[l\right]}=\left\{\begin{array}{cc} 1 & \;\text{if}\;i\to j\;\text{in layer}\;l\\ 0 & \;\text{else} \end{array}\right..$$

Then we calculate the in- and out-degree of each node in each layer

[12] $${\tilde{d}}_{1}^{[l],\text{in}}=\sum_{j}A_{ji}^{\left[l\right]},$$

[13] $${\tilde{d}}_{i}^{[l],\;\text{out}}=\sum_{j}A_{ij}^{\left[l\right]},$$

where $d_{i}^{[l],\;\text{in}},d_{i}^{[l],\;\text{out}}$ are the in-and out-degree of node i in layer l respectively.

For a k−tuple $\left(n_{1},\ldots ,n_{k}\right)$, we calculate $P_{\;\text{in}}^{\left(n_{1},\ldots ,n_{k}\right)}\left(d_{1},\ldots ,d_{k}\right)$ by counting the number of nodes i, across all samples, where

[14] $${\tilde{d}}_{i}^{[l],\;\text{in}}=d_{l},$$

for each l∈{1,…,k} simultaneously and for $d_{l}\in \left\{1,\ldots ,d_{\text{max}}\right\}$, where $d_{\text{max}}$ is the maximum degree of a node in the multilayer graph, and then dividing through by the total number of nodes in all samples. We calculate the same for $P_{\text{out}}^{\left(n_{1},\ldots ,n_{k}\right)}\left(d_{1},\ldots ,d_{k}\right)$.

As we are using a finite number of samples, we then perform distribution smoothing (81) to eliminate zeros in the empirical distribution. Instead of using,

[15] $$P^{\left(n_{1},\ldots ,n_{k}\right)}\left(d_{1},\ldots ,d_{k}\right)=\frac{N}{M},$$

where N is the number of nodes satisfying condition [**14**] and M is the total number of nodes across samples, we average the empirical distribution with a uniform prior via the following replacement,

[16] $$P^{\left(n_{1},\ldots ,n_{k}\right)}\left(d_{1},\ldots ,d_{k}\right)=\frac{1}{2}\frac{N}{M}+\frac{1}{2}\frac{1}{d_{\text{max}}^{k}}.$$

### Computing Jensen–Shannon Divergence.

We quantify the divergence between the in- and out-degree distributions using JSD which is a symmetrized version of KLD that does not suppose a model–data relationship (82). This is defined between two probability distributions P,Q as

[17] $$J\left(P|Q\right)=\frac{1}{2}D\left(P|M\right)+\frac{1}{2}D\left(Q|M\right),$$

where $M=\frac{1}{2}\left(P+Q\right)$ is an averaged distribution and D(·) represents the KLD, given by,

[18] $$\begin{array}{cc} D(P|Q) & =\sum_{x\in X}P\left(x\right)\log\frac{P(x)}{Q(x)}. \end{array}$$

As X represents the support of the distribution, it takes the form ${\left\{1,\ldots ,d_{\text{max}}\right\}}^{k}$, where k is the dimension of the probability distributions and $d_{\text{max}}$ is the maximum degree of a node in the multilayer graph. For computational feasibility, $d_{\text{max}}$ can be limited during the calculation of JSD, truncating the sum. For 5-order analysis, we limit $d_{\text{max}}$ to 75. For a systematic analysis of the effect of degree limiting see *SI Appendix*.

### MEG Data.

#### Participants.

The participant cohort consisted of 83 healthy volunteers made up of 33 males and 50 females with ages in the range 18 to 63 and a mean age of 28.76 ± 8.06. The 51 participants included in this analysis included 22 males and 29 females with ages in the range 18 to 63 and a mean age of 27.57 ± 7.13. Participants were recruited in Denmark, came from Western countries, reported normal hearing and gave informed consent before the experiment. The project was approved by the Institutional Review Board (IRB) of Aarhus University (case number: DNC-IRB-2020-006) and experimental procedures complied with the Declaration of Helsinki—Ethical Principles for Medical Research. After preprocessing, the 51 participants with at least 15 nondiscarded trials in the first experimental condition were included in the analysis. Only trials where participants correctly identified the sequence were included. For those participants with more than 15 trials, 15 trials were randomly sampled.

#### Experimental stimuli and design.

We employed an old/new paradigm auditory recognition task (33, 35, 36, 38). Participants listened to a short musical piece twice and asked to memorize it to the best of their ability. The piece was the first four bars of the right-hand part of Johann Sebastian Bach’s Prelude No. 2 in C Minor, BWV 847. Next, participants listened to 135 five-tone musical sequences, corresponding to 27 trials in 5 experimental conditions, of 1750 ms each and were requested to indicate if the sequence belonged to the original music or was a variation. Differences between experimental conditions have been described in detail by Bonetti et al (33). We consider one experimental condition, where participants recognized the original, previously memorized sequences.

#### Data acquisition.

MEG recordings were taken in a magnetically shielded room at Aarhus University Hospital, Aarhus, Denmark using an Elekta Neuromag TRIUX MEG scanner with 306 channels (Elekta Neuromag, Helsinki, Finland). The sampling rate was 1,000 Hz with analogue filtering of 0.1 to 330 Hz. For further details on the data acquisition see *SI Appendix*.

#### MEG preprocessing.

First, raw MEG sensor data were processed by MaxFilter (83) to attenuate external interferences. We then applied signal space separation (for parameters see *SI Appendix*). Then, the data were converted into Statistical Parametric Mapping (SPM) format, preprocessed and analyzed in MATLAB (MathWorks, Natick, MA, USA) using in-house codes and the Oxford Centre for Human Brain Activity (OHBA) Software Library (OSL) (84). The continuous MEG data were visually inspected and large artifacts were removed using OSL. Less than 0.1% of the collected data was removed. Next, independent component analysis (ICA) was implemented to discard artifacts in the brain data from heart-beats and eye-blinks (for details, see *SI Appendix*) (85). Last, the signal was epoched in 135 trials, 27 trials for each of 5 experimental conditions and the mean signal recorded in the baseline (the poststimulus brain signal) was removed. Each resulting trial lasted 4400 ms plus 100 ms of baseline time.

#### Source reconstruction.

We employed the beamforming method to spatially localize the MEG signal (86). For details on the beamforming algorithm and the implementation see *SI Appendix*.

## Supplementary Material

Appendix 01 (PDF)

## Data Availability

The code used to implement the DiMViGI framework is available at https://github.com/rnartallo/multilevelirreversibility (74). The in-house code used for MEG preprocessing is available at https://github.com/leonardob92/LBPD-1.0 (75). The preprocessed MEG recordings used in this analysis are freely available at https://doi.org/10.5281/zenodo.13939016 (76).

## References

[1] D. S. Bassett, O. Sporns, Network neuroscience. Nat. Neurosci. **20**, 353–364 (2017). 28230844 10.1038/nn.4502 PMC5485642

[2] C. W. Lynn, D. S. Bassett, The physics of brain network structure, function and control. Nat. Rev. Phys. **1**, 318–332 (2019).

[3] D. R. Chialvo, Emergent complex neural dynamics. Nat. Phys. **6**, 744–750 (2010).

[4] C. W. Lynn, E. J. Cornblath, L. Papadopoulos, D. S. Bassett, Broken detailed balance and entropy production in the human brain. Proc. Natl. Acad. Sci. U.S.A. **118**, e2109889118 (2021). 34789565 10.1073/pnas.2109889118 PMC8617485

[5] G. Deco, Y. Sanz-Perl, H. Bocaccio, E. Tagliazucchi, M. L. Kringelbach, The INSIDEOUT framework provides precise signatures of the balance of intrinsic and extrinsic dynamics in brain states. Commun. Biol. **5**, 572 (2022). 35688893 10.1038/s42003-022-03505-7 PMC9187708

[6] G. Deco , The arrow of time of brain signals in cognition: Potential intriguing role of parts of the default mode network. Netw. Neurosci. **7**, 966–998 (2023). 37781151 10.1162/netn_a_00300 PMC10473271

[7] R. Nartallo-Kaluarachchi , Broken detailed balance and entropy production in directed networks. Phys. Rev. E **110**, 069901 (2024). 39425339 10.1103/PhysRevE.110.034313

[8] T. Bolton, D. Van De Ville, E. Amico, M. Preti, R. Liégeois, The arrow-of-time in neuroimaging time series identifies causal triggers of brain function. Hum. Brain Mapp. **44**, 4077–4087 (2023). 37209360 10.1002/hbm.26331 PMC10258533

[9] G. Deco, C. Lynn, Y. Sanz-Perl, M. L. Kringelbach, Violations of the fluctuation-dissipation theorem reveal distinct non-equilibrium dynamics of brain states. Phys. Rev. E **108**, 064410 (2023). 38243472 10.1103/PhysRevE.108.064410

[10] M. Gilson, E. Tagliazucchi, R. Cofré, Entropy production of multivariate Ornstein-Uhlenbeck processes correlates with consciousness levels in the human brain. Phys. Rev. E **107**, 024121 (2023). 36932548 10.1103/PhysRevE.107.024121

[11] E. Guzmán , The lack of temporal brain dynamics asymmetry as a signature of impaired consciousness states. Interface Focus **13**, 20220086 (2023). 37065259 10.1098/rsfs.2022.0086 PMC10102727

[12] J. Cruzat , Temporal irreversibility of large-scale brain dynamics in Alzheimer’s disease. J. Neurosci. **43**, 1643–1656 (2023). 36732071 10.1523/JNEUROSCI.1312-22.2022 PMC10008060

[13] L. de la Fuente , Temporal irreversibility of neural dynamics as a signature of consciousness. Cereb. Cortex **33**, 1856–1865 (2023). 35512291 10.1093/cercor/bhac177

[14] S. Carnot, Réflexions sur la puissance motrice du feu et sur les machines propres à développer cette puissance (Bachelier, Paris, 1824).

[15] R. Clausius, Ueber die bewegende kraft der wärme und die gesetze, welche sich daraus für die wärmelehre selbst ableiten lassen. Ann. Phys. **79**, 368–397 (1850).

[16] C. Jarzynski, Nonequilibrium equality for free energy differences. Phys. Rev. Lett. **78**, 2690 (1997).

[17] G. E. Crooks, Entropy production fluctuation theorem and the nonequilibrium work relation for free energy differences. Phys. Rev. E **60**, 2721 (1999). 10.1103/physreve.60.2721 11970075

[18] R. K. P. Zia, B. Schmittmann, Probability currents as principal characteristics in the statistical mechanics of non-equilibrium steady states. J. Stat. Mech Theory Exp. **2007**, P07012 (2007).

[19] A. S. Eddington, The Nature of the Physical World (Cambridge University Press, 1928).

[20] U. Seifert, Stochastic thermodynamics, fluctuation theorems and molecular machines. Rep. Progress Phys. **75**, 126001 (2012). 10.1088/0034-4885/75/12/126001 23168354

[21] U. Seifert, Entropy production along a stochastic trajectory and an integral fluctuation theorem. Phys. Rev. Lett. **95**, 040602 (2005). 16090792 10.1103/PhysRevLett.95.040602

[22] R. Kawai, J. M. R. Parrondo, C. V. den Broeck, Dissipation: The phase-space perspective. Phys. Rev. Lett. **98**, 080602 (2007). 17359081 10.1103/PhysRevLett.98.080602

[23] E. H. Feng, G. E. Crooks, Length of time’s arrow. Phys. Rev. Lett. **101**, 090602 (2008). 18851595 10.1103/PhysRevLett.101.090602

[24] E. Roldán, J. M. R. Parrondo, Estimating dissipation from single stationary trajectories. Phys. Rev. Lett. **105**, 150607 (2010). 21230886 10.1103/PhysRevLett.105.150607

[25] E. Roldán, Irreversibility and Dissipation in Microscopic Systems (Springer, 2014).

[26] M. L. Kringelbach, Y. Sanz-Perl, E. Tagliazucchi, G. Deco, Toward naturalistic neuroscience: Mechanisms underlying the flattening of brain hierarchy in movie-watching compared to rest and task. Sci. Adv. **9**, 36638163 (2023). 10.1126/sciadv.ade6049 PMC9839335 36638163

[27] G. Deco, D. Vidaurre, M. L. Kringelbach, Revisiting the global workspace orchestrating the hierarchical organization of the human brain. Nat. Hum. Behav. **5**, 497–511 (2021). 33398141 10.1038/s41562-020-01003-6 PMC8060164

[28] C. W. Lynn, C. M. Holmes, W. Bialek, D. J. Schwab, Decomposing the local arrow of time in interacting systems. Phys. Rev. Lett. **129**, 118101 (2022). 36154397 10.1103/PhysRevLett.129.118101 PMC9751844

[29] C. W. Lynn, C. M. Holmes, W. Bialek, D. J. Schwab, Emergence of local irreversibility in complex interacting systems. Phys. Rev. E **106**, 034102 (2022). 36266789 10.1103/PhysRevE.106.034102 PMC9751845

[30] D. Sekizawa, S. Ito, M. Oizumi, Decomposing thermodynamic dissipation of linear Langevin systems via oscillatory modes and its application to neural dynamics. Phys. Rev. X 14 (2024).

[31] L. Lacasa, B. Luque, F. Ballesteros, J. Luque, J. C. Nuño, From time series to complex networks: The visibility graph. Proc. Natl. Acad. Sci. U.S.A. **105**, 4972–4975 (2008). 18362361 10.1073/pnas.0709247105 PMC2278201

[32] L. Lacasa, A. Nuñez, E. Roldán, J. Parrondo, B. Luque, Time series irreversibility: A visibility graph approach. Eur. Phys. J. B. **85**, 217 (2012).

[33] L. Bonetti , Spatiotemporal brain hierarchies of auditory memory recognition and predictive coding. Nat. Commun. **15**, 4313 (2024). 38773109 10.1038/s41467-024-48302-4 PMC11109219

[34] L. Bonetti , Brain recognition of previously learned versus novel temporal sequences: A differential simultaneous processing. Cereb. Cortex **33**, 5524–5537 (2023). 36346308 10.1093/cercor/bhac439 PMC10152090

[35] L. Bonetti , Age-related neural changes underlying long-term recognition of musical sequences. Commun. Biol. **7**, 1036 (2024). 39209979 10.1038/s42003-024-06587-7 PMC11362492

[36] G. Fernández-Rubio , Magnetoencephalography recordings reveal the spatiotemporal dynamics of recognition memory for complex versus simple auditory sequences. Commun. Biol. **5**, 1272 (2022). 36402843 10.1038/s42003-022-04217-8 PMC9675809

[37] G. Fernández-Rubio , Investigating the impact of age on auditory short-term, long-term, and working memory. Psychol. Music **52**, 187–198 (2024).

[38] G. Fernández-Rubio, F. Carlomagno, P. Vuust, M. L. Kringelbach, L. Bonetti, Associations between abstract working memory abilities and brain activity underlying long-term recognition of auditory sequences. PNAS Nexus **1**, 1–10 (2022). 10.1093/pnasnexus/pgac216 PMC9802106 36714830

[39] L. Bonetti , Spatiotemporal whole-brain activity and functional connectivity of melodies recognition. Cereb. Cortex **34**, bhae320 (2024). 39110413 10.1093/cercor/bhae320 PMC11304985

[40] L. Bonetti , The neural mechanisms of concept formation over time in music. bioRxiv [Preprint] (2024). 10.1101/2024.11.06.622228 (Accessed 2 January 2025).

[41] L. Bonetti , BROADband brain Network Estimation via Source Separation (BROAD-NESS). bioRxiv [Preprint] (2024). 10.1101/2024.10.31.621257 (Accessed 2 January 2025).

[42] L. Bonetti , Working memory predicts long-term recognition of auditory sequences: Dissociation between confirmed predictions and prediction errors. bioRxiv [Preprint] (2024). 10.1101/2024.09.20.614110 (Accessed 2 January 2025). PMC12611408 40400073

[43] T. F. Varley, O. Sporns, Network analysis of time series: Novel approaches to network neuroscience. Front. Neurosci. **15**, 787068 (2022). 35221887 10.3389/fnins.2021.787068 PMC8874015

[44] S. Sulaimany, Z. Safahi, Visibility graph analysis for brain: Scoping review. Front. Neurosci. **17**, 1268485 (2023). 37841678 10.3389/fnins.2023.1268485 PMC10570536

[45] A. M. Nunez, L. Lacasa, J. P. Gomez, B. Luque, “Visibility algorithms: A short review” in New Frontiers in Graph Theory, Y. Zhang, Ed. (InTech, 2012), pp. 119–152.

[46] L. Lacasa, V. Nicosia, V. Latora, Network structure of multivariate time series. Sci. Rep. **5**, 15508 (2015). 26487040 10.1038/srep15508 PMC4614448

[47] J. F. Donges, R. V. Donner, J. Kurths, Testing time series reversibility using complex network methods. Europhys. Lett. **102**, 10004 (2012).

[48] G. Weiss, Time-reversibility of linear stochastic processes. J. Appl. Probab. **12**, 831–836 (1975).

[49] G. Deco , One ring to rule them all: The unifying role of prefrontal cortex in steering task-related brain dynamics. Prog. Neurobiol. **227**, 102468 (2023). 37301532 10.1016/j.pneurobio.2023.102468

[50] K. Friston, Predictive coding, precision and synchrony. Cogn. Neurosci. **3**, 238–239 (2012). 24171746 10.1080/17588928.2012.691277

[51] S. Koelsch, P. Vuust, K. Friston, Predictive processes and the peculiar case of music. Trends Cogn. Sci. **23**, 63–77 (2019). 30471869 10.1016/j.tics.2018.10.006

[52] P. Vuust, O. A. Heggli, K. J. Friston, M. L. Kringelbach, Music in the brain. Nat. Rev. Neurosci. **23**, 287–305 (2022). 35352057 10.1038/s41583-022-00578-5

[53] K. Friston, A theory of cortical responses. Philos. Trans. R. Soc. B **360**, 815–836 (2005). 10.1098/rstb.2005.1622 PMC1569488 15937014

[54] K. Friston, Hierarchical models in the brain. PLoS Comput. Biol. **4**, e1000211 (2008). 18989391 10.1371/journal.pcbi.1000211 PMC2570625

[55] K. Friston, S. Kiebel, Predictive coding under the free-energy principle. Philos. Trans. R. Soc. B **364**, 1211–1221 (2009). 10.1098/rstb.2008.0300 PMC2666703 19528002

[56] T. A. Hackett, Information flow in the auditory cortical network. Hear. Res. **271**, 133–146 (2011). 20116421 10.1016/j.heares.2010.01.011 PMC3022347

[57] E. M. Rouiller, G. M. Simm, A. E. Villa, Y. de Ribaupierre, F. de Ribaupierre, Auditory corticocortical interconnections in the cat: evidence for parallel and hierarchical arrangement of the auditory cortical areas. Exp. Brain Res. **86**, 483–505 (1991). 1722171 10.1007/BF00230523

[58] A. J. E. Kell, D. L. K. Yamins, E. N. Shook, S. V. Norman-Haignere, J. H. McDermott, A task-optimized neural network replicates human auditory behavior, predicts brain responses, and reveals a cortical processing hierarchy. Neuron **98**, 630–644 (2018). 29681533 10.1016/j.neuron.2018.03.044

[59] K. Okada , Hierarchical organization of human auditory cortex: Evidence from acoustic invariance in the response to intelligible speech. Cereb. Cortex **20**, 2486–2495 (2010). 20100898 10.1093/cercor/bhp318 PMC2936804

[60] M. Lumaca, B. Kleber, E. Brattico, P. Vuust, G. Baggio, Functional connectivity in human auditory networks and the origins of variation in the transmission of musical systems. eLife **8**, e48710 (2019). 31658945 10.7554/eLife.48710 PMC6819097

[61] J. H. Lestang, H. Cai, B. B. Averbeck, Y. E. Cohen, Functional network properties of the auditory cortex. Hear. Res. **433**, 108768 (2023). 37075536 10.1016/j.heares.2023.108768 PMC10205700

[62] G. Petri , Homological scaffolds of brain functional networks. J. R. Soc. Interface **11**, 20140873 (2014). 25401177 10.1098/rsif.2014.0873 PMC4223908

[63] A. Santoro, F. Battiston, G. Petri, E. Amico, Higher-order organization of multivariate time series. Nat. Phys. **19**, 221–229 (2023).

[64] M. R. R. Tabar , Revealing higher-order interactions in high-dimensional complex systems: A data-driven approach. Phys. Rev. X **14**, 011050 (2024).

[65] R. F. Betzel, J. Faskowitz, O. Sporns, Living on the edge: Network neuroscience beyond nodes. Trends Cogn. Sci. **27**, 1068–1084 (2023). 37716895 10.1016/j.tics.2023.08.009 PMC10592364

[66] C. Giusti, R. Ghrist, D. S. Bassett, Two’s company, three (or more) is a simplex. J. Comput. Neurosci. **41**, 1–14 (2016). 27287487 10.1007/s10827-016-0608-6 PMC4927616

[67] T. F. Varley, M. Pope, M. G. Puxeddu, J. Faskowitz, O. Sporns, Partial entropy decomposition reveals higher-order information structures in human brain activity. Proc. Natl. Acad. Sci. U.S.A. **120**, e2300888120 (2023). 37467265 10.1073/pnas.2300888120 PMC10372615

[68] A. I. Luppi , A synergistic core for human brain evolution and cognition. Nat. Neurosci. **25**, 771–782 (2022). 35618951 10.1038/s41593-022-01070-0 PMC7614771

[69] A. I. Luppi, F. E. Rosas, P. A. Mediano, D. K. Menon, E. A. Stamatakis, Information decomposition and the informational architecture of the brain. Trends Cogn. Sci. **28**, 352–368 (2024). 38199949 10.1016/j.tics.2023.11.005

[70] R. Lambiotte, M. Rosvall, I. Scholtes, From networks to optimal higher-order models of complex systems. Nat. Phys. **15**, 313–320 (2019). 30956684 10.1038/s41567-019-0459-y PMC6445364

[71] F. Battiston , The physics of higher-order interactions in complex systems. Nat. Phys. **17**, 1093–1098 (2021).

[72] F. E. Rosas , Disentangling high-order mechanisms and high-order behaviours in complex systems. Nat. Phys. **18**, 476–477 (2022).

[73] S. Sannino, S. Stramaglia, L. Lacasa, D. Marinazzo, Visibility graphs for fMRI data: Multiplex temporal graphs and their modulations across resting-state networks. Netw. Neurosci. **1**, 208–221 (2017). 29911672 10.1162/NETN_a_00012 PMC5988401

[74] R. Nartallo-Kaluarachchi, DiMViGI Framework. GitHub. https://github.com/rnartallo/multilevelirreversibility. Deposited 16 October 2024.

[75] L. Bonetti, MEG Preprocessing. GitHub. https://github.com/leonardob92/LBPD-1.0. Deposited 16 October 2024.

[76] R. Nartallo-Kaluarachchi , Multilevel irreversibility reveals higher-order organisation of non-equilibrium interactions in human brain dynamics. Zenodo. 10.5281/zenodo.13939016. Deposited 16 October 2024. PMC11912438 40053364

[77] C. W. J. Granger, Investigating causal relations by econometric models and cross-spectral methods. Econometrica **37**, 424–438 (1969).

[78] T. Schreiber, Measuring information transfer. Phys. Rev. Lett. **85**, 461 (2000). 10991308 10.1103/PhysRevLett.85.461

[79] I. Z. Steinberg, On the time reversal of noise signals. Biophys. J. **50**, 171–179 (1986). 3730501 10.1016/S0006-3495(86)83449-X PMC1329669

[80] A. Crisanti, A. Puglisi, D. Villamaina, Nonequilibrium and information: The role of cross correlations. Phys. Rev. E **85**, 061127 (2012). 10.1103/PhysRevE.85.061127 23005071

[81] C. D. Manning, P. Raghavan, H. Schütze, Introduction to Information Retrieval (Cambridge University Press, 2008), p. 260.

[82] F. Nielsen, On the Jensen-Shannon symmetrization of distances relying on abstract means. Entropy **21**, 485 (2019). 33267199 10.3390/e21050485 PMC7514974

[83] S. Taulu, J. Simola, Spatiotemporal signal space separation method for rejecting nearby interference in MEG measurements. Phys. Med. Biol. **51**, 1–10 (2010). 10.1088/0031-9155/51/7/008 16552102

[84] M. Woolrich, L. Hunt, A. Groves, G. Barnes, MEG beamforming using Bayesian PCA for adaptive data covariance matrix regularization. NeuroImage **57**, 1466–1479 (2011). 21620977 10.1016/j.neuroimage.2011.04.041 PMC4894461

[85] D. Mantini , A signal-processing pipeline for magnetoencephalography resting-state networks. Brain Connectivity **1**, 49–59 (2011). 22432954 10.1089/brain.2011.0001

[86] A. Hillebrand, G. R. Barnes, Beamformer analysis of MEG data. Int. Rev.Neurobiol. **68**, 149–171 (2005). 16443013 10.1016/S0074-7742(05)68006-3

