# Supplementary material for: Multilevel irreversibility reveals higher-order organization of nonequilibrium interactions in human brain dynamics
Source: Proc Natl Acad Sci U S A. 2025 Mar 7;122(10):e2408791122. doi: 10.1073/pnas.2408791122 (PMC11912438; doi:10.1073/pnas.2408791122)
Supplement: Supplementary file 1 — Appendix 01 (PDF) [file pnas.2408791122.sapp.pdf]

Supporting Information for

Multilevel irreversibility reveals higher-order organisation of non-equilibrium interactions in human brain dynamics

Ramón Nartallo-Kaluarachchi, Leonardo Bonetti, Gemma Fernández-Rubio, Peter Vuust, Gustavo Deco, Morten L. Kringelbach, Renaud Lambiotte and Alain Goriely

Corresponding authors: Ramón Nartallo-Kaluarachchi, Alain Goriely.
E-mail: rnartallo@turing.ac.uk; goriely@maths.ox.ac.uk

This PDF file includes:

Supporting text
Figs. S1 to S15
SI References

Supporting Information Text

1. Introduction

In this supporting information, we provide additional results and analysis not included in the main manuscript. This supporting information is organised as follows. In Section 2, we show the results of the directed multiplex visibility graph irreversibility (DiMViGI) framework applied to data from individual participants to produce distributions of participant spread for the irreversibility of each tuple, rather than the cohort-level analysis presented in the main manuscript. We assess the significance of the differences between tuples using pairwise $t-$tests and one-way ANOVAs. Moreover, we calculate the correlations between the cohort and participant level analysis. In Section 3, we further validate the significance of the results obtained at the participant level by shuffling the time-series to produce surrogate data. We show that shuffling restores detailed balance and that the irreversibility of the true signals is significantly higher than the surrogate data. This indicates that the measured irreversibility is due to the non-equilibrium dynamics present in the time-series and not bias from finite data. In Section 4, we employ a second method to correct for biases stemming from 'finite' data. We use sub-sampling to estimate the irreversibility for various fractions of our data. We show that our results are robust to various sub-samplings of the data. Additionally, this produces a distribution of the irreversibility measurement, which we use to validate that the relative error in our measurement is significantly lower than the difference between tuples. Next, in Section 5, we show that the DiMViGI framework factorises for independent variables, theoretically validating the argument that it captures 'true' higher-order interactions. Using the factorisation, in Section 6, we are able to define the unique irreversibility generated by a higher order interaction by removing the lower-level interactions. We compare this to the 'combined' results presented in the manuscript. In Section 7, we validate that our method captures a correlate of the entropy production rate by using simulated data from four specific examples of the multivariate Ornstein-Uhlenbeck process. In Section 8, we investigate the effect on the results of limiting the maximum degree in the support of the distributions, an approach that improves computational efficiency whilst only minimally reducing accuracy. In Section 9, we discuss the definitions of entropy production rate for Markovian and non-Markovian dynamics. In Section 10 we present a comprehensive description of the experimental paradigm and the techniques used to record and pre-process the magnetoencephalography (MEG) data. In Section 11, we present an evaluation of the signal quality and signal to noise ratio as well as visualisations of baseline activity. Finally, in Section 12, we compare the results of applying our analysis to the full epoch, as presented in the paper and the rest of the SI, as well as an epoch with the pre-stimulus baseline removed and an epoch with both the pre-stimulus and post-task reset to baseline removed. We use quadruplets as an example, and show that the results are equivalent.

2. The DiMViGI framework applied to participant-level data

In this section we show the results of applying the DiMViGI framework to data at the participant-level and obtain distributions for the irreversibility of each tuple. As mentioned in the main manuscript, we analysed MEG recordings from 51 participants with 15 trials per participant. In the cohort-level analysis presented in the main manuscript, we constructed the in- and out-degree distributions using $51 \times 15 = 765$ samples of the multiplex network. In order to examine the spread between participants, we repeat the same analysis for each participant in isolation, using only the 15 associated trials. As a result the degree distributions are much more poorly estimated and produce much higher divergences. Nevertheless, we are able to quantify the irreversibility of each tuple of brain regions for each participant and examine the distribution.

Figure S1 shows the results of the DiMViGI analysis for the participant-level data distributions of the irreversibility for each tuple in each level. Panel a) shows the schematic representation of the 6 regions of interest (ROIs) that correspond to variables in the multivariate time-series. The icons on the x-axis of the subsequent panels indicate which ROIs are included in each tuple. Panels b-f) show the participant-level distributions for 1-5 order respectively. We run one-way ANOVAs and find that, at each level 1-5, the tuple is a significant predictor of irreversibility ($p < 0.00001$). In addition, we run paired $t-$tests to see which tuples at each level are significantly different in a pairwise comparison applying a Bonferroni correction for multiple comparisons at each level k (1). Figure S2 displays the significance results of the pairwise $t-$tests. The corrected significance of each comparison is denoted as follows: (ns) if $p > 0.05$; (*) if $p < 0.05$; (**) if $p < 0.01$; (***) if $p < 0.001$ and (****) if $p < 0.0001$. Figure S2 shows that at level 1, the difference between each tuple in pairwise comparison is significant ($p < 0.0001$). In addition, it shows that at levels 2-5, there is a mixture of significant and not significant differences depending on the number of ROIs in common between the compared tuples.

Finally, we compare the participant-level analysis to the cohort-level analysis by calculating the ranking of tuples at each level for each participant and comparing it to the cohort-level ranking, using Spearman's ρ. In addition, for each participant at each level, we calculate Pearson's r (correlation coefficient) between the participant level and the cohort level. Panel a) of Fig S3 shows the ρ for each participant at each level when compared to the cohort-level ranking. Panel b) of Fig S3 shows the r for each participant at each level when compared to the cohort-level measurements. Both show that at lower orders (1-3), the measurements, and rankings, obtained from the participants in isolation agree closely with the cohort-level results. However, at higher order (4-5), the low number of samples, 15, in the participant-level analysis is not enough to accurately estimate the high-dimensional degree distributions leading to a lack of agreement between the well-estimated cohort analysis and the poorly-estimated participant analysis.

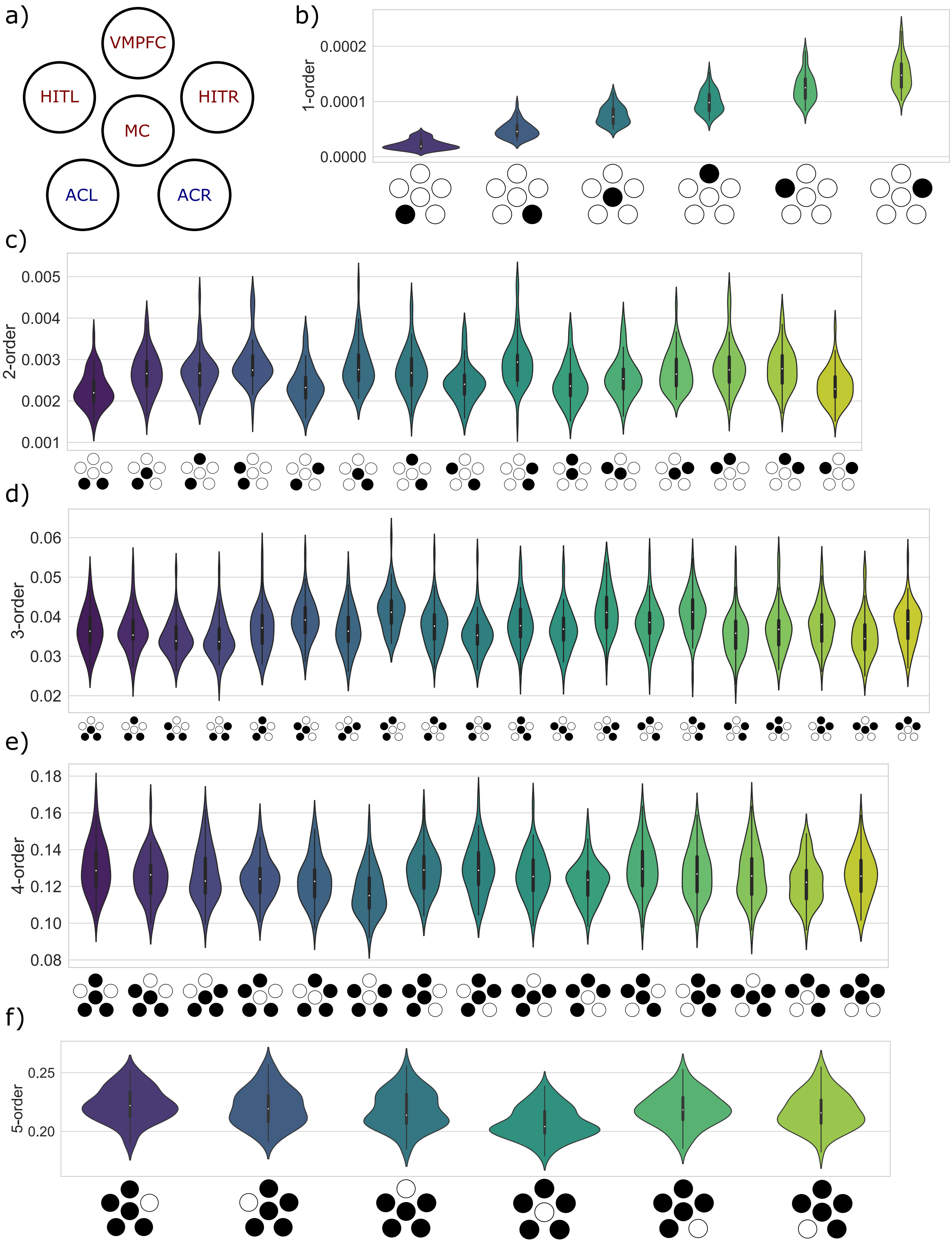

Fig. S1. Participant distribution of irreversibility for each tuple. a) Schematic representation of the 6 brain regions of interest (ROIs) in the MEG recordings. The icon in the following panels indicates which regions are included in each tuple. b) 1-order irreversibility distribution for each ROI in isolation. The results follow the same hierarchy as the cohort-level analysis in the main manuscript. c) 2-order irreversibility distribution for each pairs of ROIs. d) 3-order irreversibility distribution for each triplet of ROIs. e) 4-order irreversibility distribution for each quadruplet of ROIs. f) 5-order irreversibility distribution for each quintuplet of ROIs.

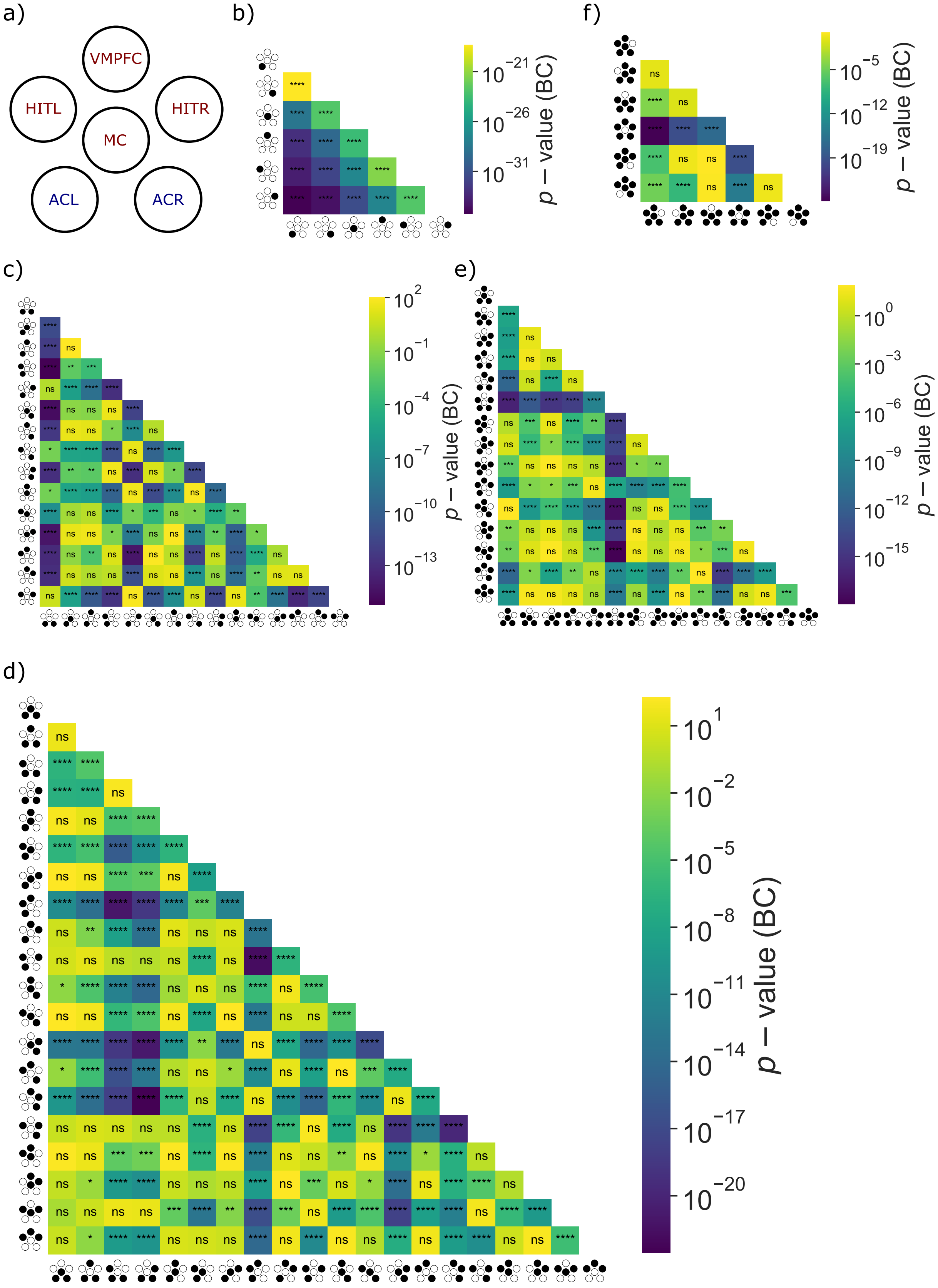

Fig. S2. Results of pairwise $t-$tests (paired) between all pairs of k-tuples at each order k with a Bonferroni correction for multiple comparisons (1). The corrected significance of each comparison is denoted as follows: (ns) if $p > 0.05$; (*) if $p < 0.05$; (**) if $p < 0.01$; (***) if $p < 0.001$ and (****) if $p < 0.0001$. Panel a) shows a schematic representation of the 6 brain regions of interest (ROIs) in the MEG recordings. The remaining panels show the results for b) singletons, c) pairs, d) triplets, e) quadruplets, f) quintuplets. There is a mixture of significant and not significant differences depending on the number of ROIs in common between the compared tuples

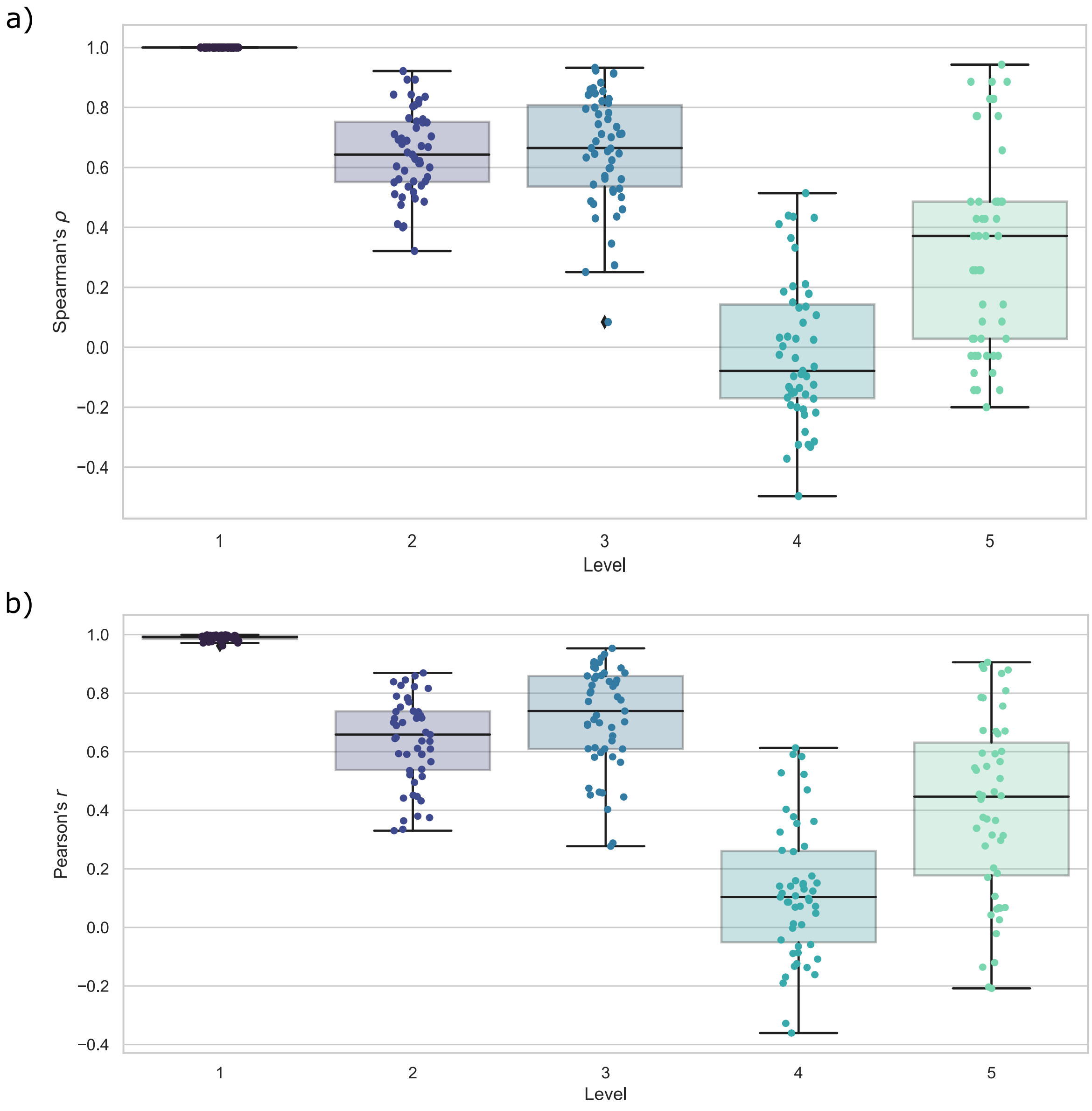

Fig. S3. Correlations between participant and cohort level results. a) Spearman's ρ coefficient for the ranking of tuples at each level for each participant when compared to the ranking obtained from the cohort-level analysis. b) Pearson's r correlation coefficient for the measurements of tuples at each level for each participant when compared to the measurements obtained from the cohort-level analysis. The figure shows that at lower orders, the participant distributions agree more closely with the cohort-level analysis. However, at higher orders, the degree distributions are more poorly estimated leading to low agreement between the cohort and participant-level analysis.

3. Validation of results against surrogate data from shuffling time-series

When measuring information-theoretic quantities from finite-length series, a bias is introduced. Finite-length time-series have a level of irreversibility that emerges from their finiteness as opposed to the temporal organisation of the data (2, 3). In order to validate that the measured irreversibilities are significant and therefore emerge from the temporal structure of the data, we must compare them to surrogate data. When generating surrogate data, we aim to break the temporal correlations and restore detailed balance. In order to do this, we randomly shuffled the time-series in time. This means that the number of occurrences of each state remains the same as the original data, but the sequence of states is now randomised thereby restoring detailed balance, as shown by Lynn et al (3). The irreversibility measured in the shuffled time-series is an estimate of the 'noise-floor' which stems from finiteness of the series. If the irreversibility of the true signal is significantly higher than the noise floor, then this is due to the system violating detailed balance in the underlying dynamics.

Other common approaches for generating surrogate time-series such as phase randomisation or Fourier transform surrogates preserve the temporal structure of the time-series and are therefore unsuitable for this application (4, 5).

Figure S4 shows the comparison of the measurements in the original MEG time-series and its randomly shuffled surrogate. For each tuplet the difference between the shuffled and original time-series is significant ($p < 0.0001$). This shows that the irreversibility measured using the DiMViGI framework is a significant statistical feature of the multivariate time-series as the shuffled data is measured to be far more reversible using the DiMViGI framework.

4. Estimating finite-data errors using sub-sampling

In addition to shuffling the time-series, another approach for estimating errors that arise from finite-data, is to employ a sub-sampling approach (2, 3, 6). As the DiMViGI framework, when applied to all participants, calculates a single quantity for the irreversibility of each tuple, we need to estimate the size of the error as well as how this error evolves with the amount of data that is available. To do this, we consider the relationship between irreversibility and the *inverse-data-fraction* (IDF) which is 1 divided by the fraction of the data that is used to estimate the irreversibility. We do so by randomly sampling (without replacement) 12 and then 9 trials per participant in a hierarchical fashion, meaning 9 are chosen from the 12 which are chosen from the 15. Whilst keeping the contribution of each participant equal, this allows us to calculate the irreversibility using fractions 1, 0.8, 0.6 and of the full dataset corresponding to IDF=1, 1.25 and 1.67 respectively. Importantly, there are many ways to sub-sample the data which allows one to obtain a number of different estimates, and thus an uncertainty measure, for the irreversibility at IDF=1.25 and 1.67. In the following analysis, we use 20 random sub-samplings. Some studies in neural spike trains linearly extrapolate this to the *infinite data limit*, corresponding to IDF=0 (6–10). However, we find that the relationship between irreversibility under DiMViGI and IDF is nonlinear. Therefore this extrapolation may yield nonphysical (negative) values of irreversibility and thus is not suited for our analysis. We note that such analysis is often useful when attempting to measure the *true* value of a quantity in particular units, for comparison to other experiments. In our study, the measurements obtained by DiMViGI are relative quantities, to be compared within a given level k.

Figure S5 shows the results of sub-sampling at order 1. Panels a) and c) show that the 1-order irreversibilities measured at IDF = 1.25 and 1.67 agree closely with IDF =1, the results in the main manuscript. Furthemore, panels b) and d) show the pairwise comparisons between the distributions of irreversibility measured for each ROI at both IDF= 1.25 and 1.67 (paired $t-$test, BC). We find each comparison is significant (****, $p < 0.0001$) indicating that the differences between ROIs are much larger than the errors in the measured irreversibility. Figures S6, S7 show similar results at order 2, pairs of regions, and order 3, triplets, where pairwise comparison shows a range of significant differences. Figures S8 & S9 show that the results for orders 4 & 5, quadruplets and quintuplets, agree between IDF=1 and IDF=1.25, 1.67. Furthermore, we find that most quadruplets and all quintuplets have significantly different levels of irreversibility. These results suggest that biases and errors in the irreversibility measurements are typically smaller than differences in measurements between tuples and further validates our approach of identifying particularly (ir)reversible tuples. We note that the quintuplet analysis for the sub-sampling uses degree-limiting with a maximum degree of 45, which, as shown in Section 8, minimally affects the results whilst improving the computational efficiency.

5. The DiMViGI framework factorises for independent variables

To illustrate that the DiMViGI framework indeed can differentiate higher order interactions from the composition of lower order ones, we consider the framework applied to a k-tuple of variables, $(x_1, ...x_k)$. First we assume that x_1 is independent of $(x_2, ..., x_k)$ and show that we can write the irreversibility of the $k-$tuple as the sum of the irreversibility of x_1 plus the irreversibility of the $(k-1)-$tuple $(x_2, ..., x_k)$. Inductively, we can show that the DiMViGI framework factorises for independent variables meaning that the irreversibility of their interaction is merely the sum of the irreversibility of each variable in isolation. This validates that the framework is truly capturing multilevel irreversibility.

Consider $(x_1, ..., x_k)$ such that x_1 is independent of the other variables. As x_1 is independent, the edges in the associated layer of the multiplex network are also independent. As a result, the joint (in- and out-) degree distribution of the

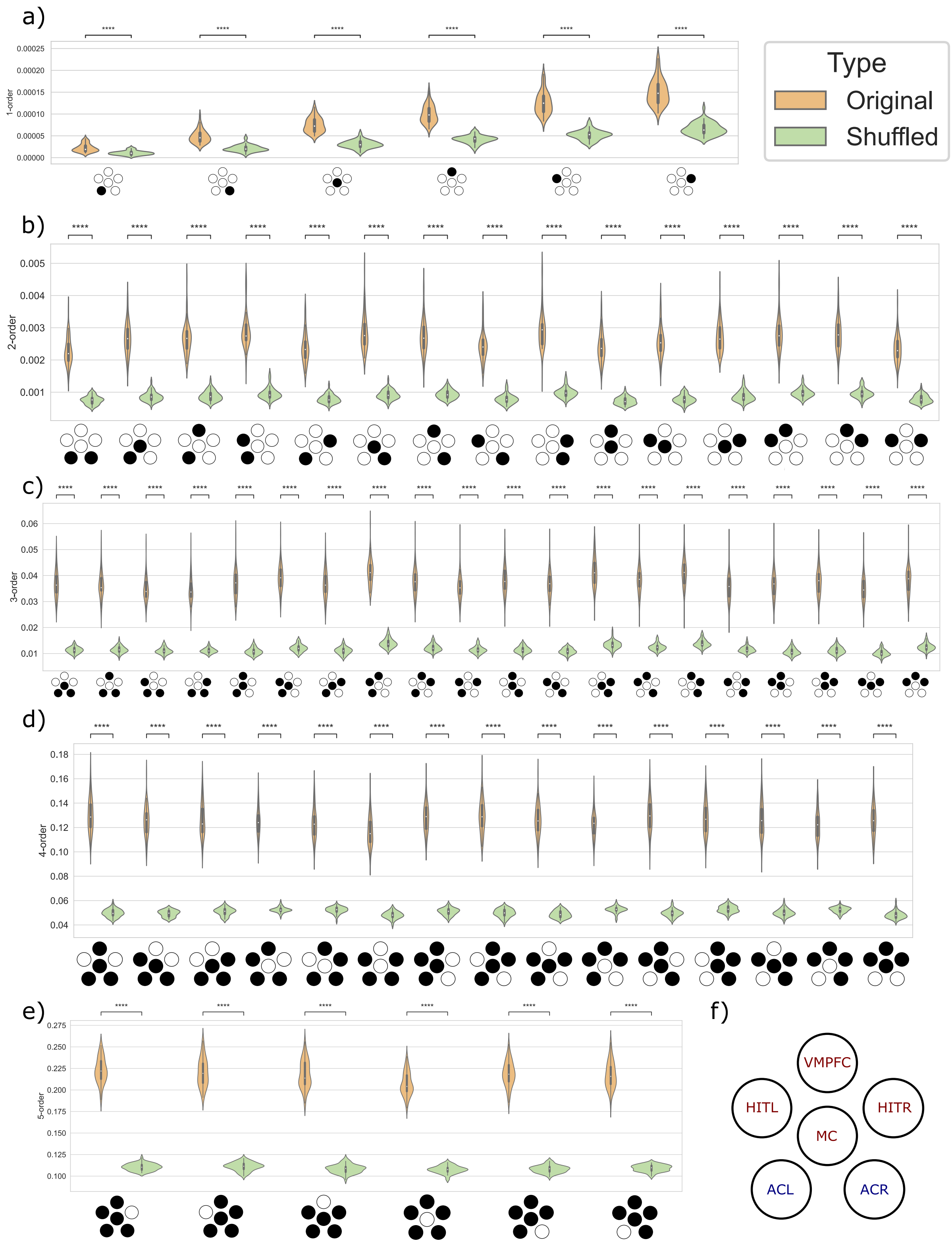

Fig. S4. Comparison of original neural recording against surrogate data obtained via shuffling the time-series in time. The difference between the original and shuffled data for each tuple at each level is significant. We label (****) if $p < 0.0001$. a) 1-order irreversibility is significant ($p < 0.0001$) for each ROI when compared to shuffled data. b) 2-order irreversibility is significant ($p < 0.0001$) for each pair of ROIs when compared to shuffled data. c) 3-order irreversibility is significant ($p < 0.0001$) for each triplet of ROIs when compared to shuffled data. d) 4-order irreversibility is significant ($p < 0.0001$) for each quadruplet of ROIs when compared to shuffled data. e) 5-order irreversibility is significant ($p < 0.0001$) for each quintuplet of ROIs when compared to shuffled data. f) Schematic representation of the 6 brain regions of interest (ROIs) in the MEG recordings. The icon in the preceding panels indicates which regions are included in each tuple.

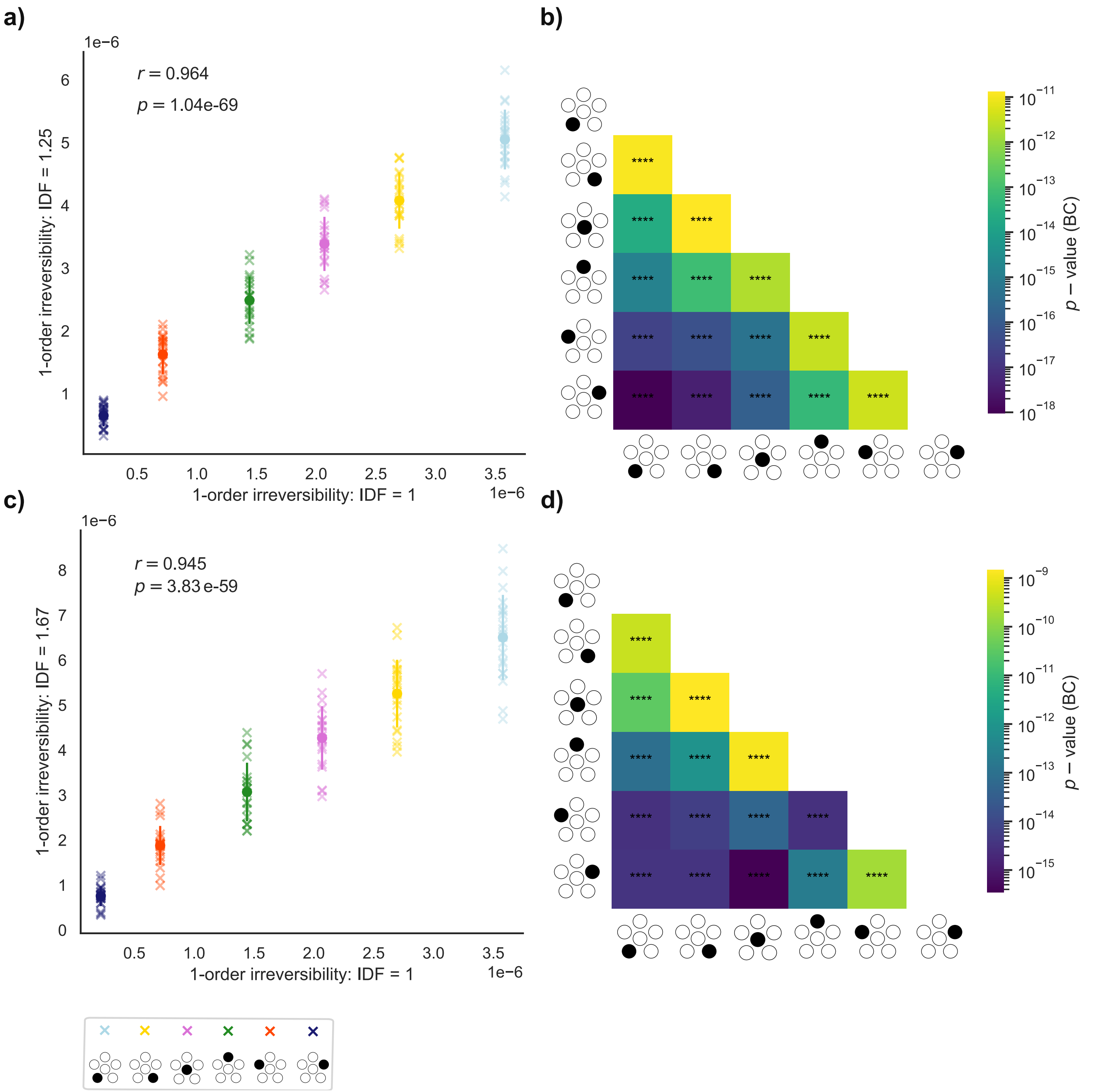

Fig. S5. Sub-sampling for estimation of finite-data errors at order 1. a) A comparison between the original results at IDF=1 and the results at 20 samples at IDF=1.25 (12/15 trials). We find that the results are highly correlated indicating that our method is robust to different amounts of data. b) Bonferroni-corrected paired $t-$tests show that each pairwise comparison is significant (****,$p < 0.0001$), indicating that the differences in irreversibilities are far beyond noise-level. c) A comparison between the original results at IDF=1 and the results at 20 samples at IDF=1.67 (9/15 trials). We find that the results are highly correlated. d) Bonferroni-corrected paired $t-$tests show that each pairwise comparison is significant (****,$p < 0.0001$).

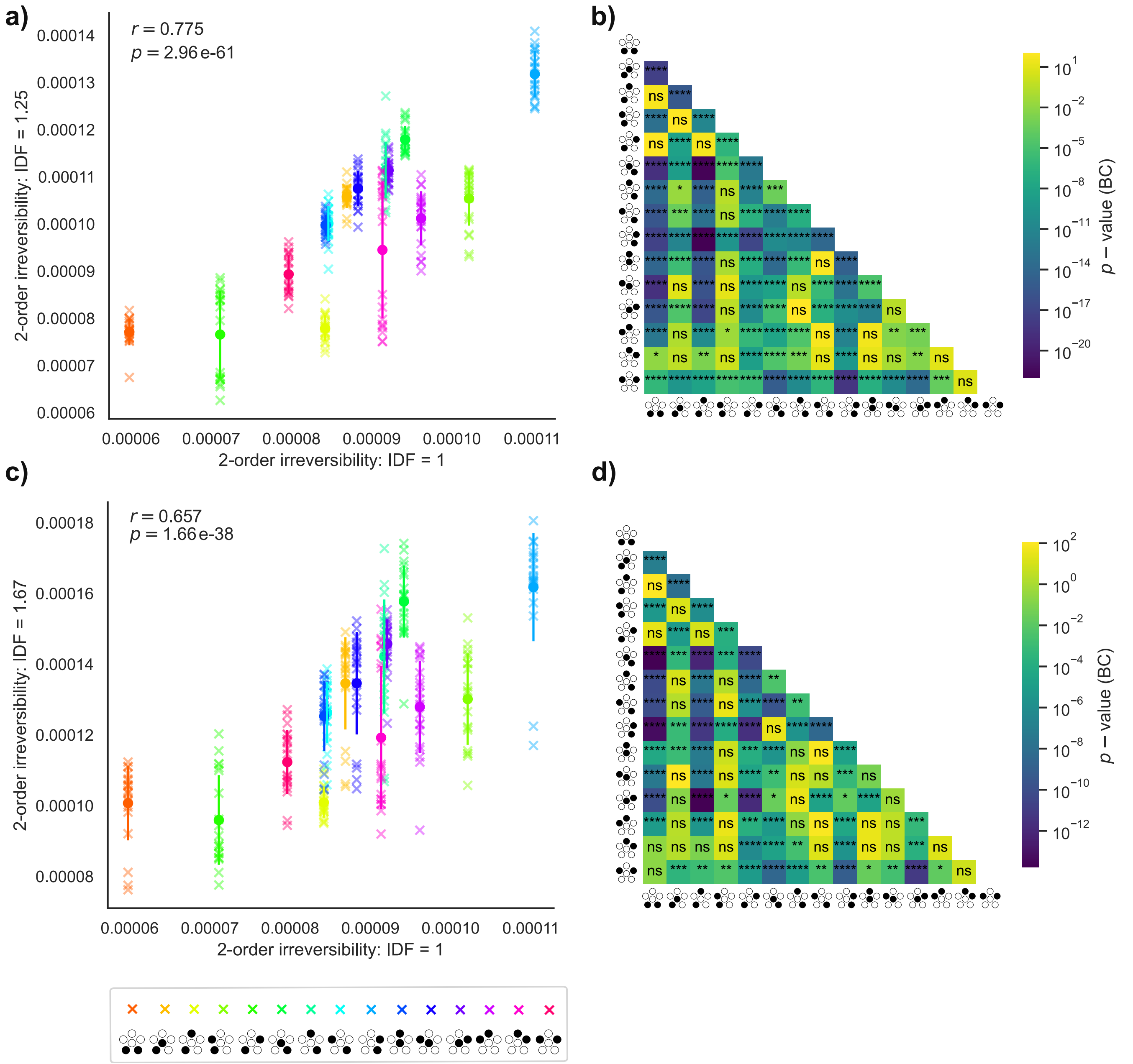

Fig. S6. Sub-sampling for estimation of finite-data errors at order 2. a) A comparison between the original results at IDF=1 and the results at 20 samples at IDF=1.25 (12/15 trials). We find that the results are highly correlated. b) Bonferroni-corrected paired $t-$tests show a range of significant pairwise comparisons. c) A comparison between the original results at IDF=1 and the results at 20 samples at IDF=1.67 (9/15 trials). We find that the results are highly correlated. d) Bonferroni-corrected paired $t-$tests show a range of significant pairwise comparisons.

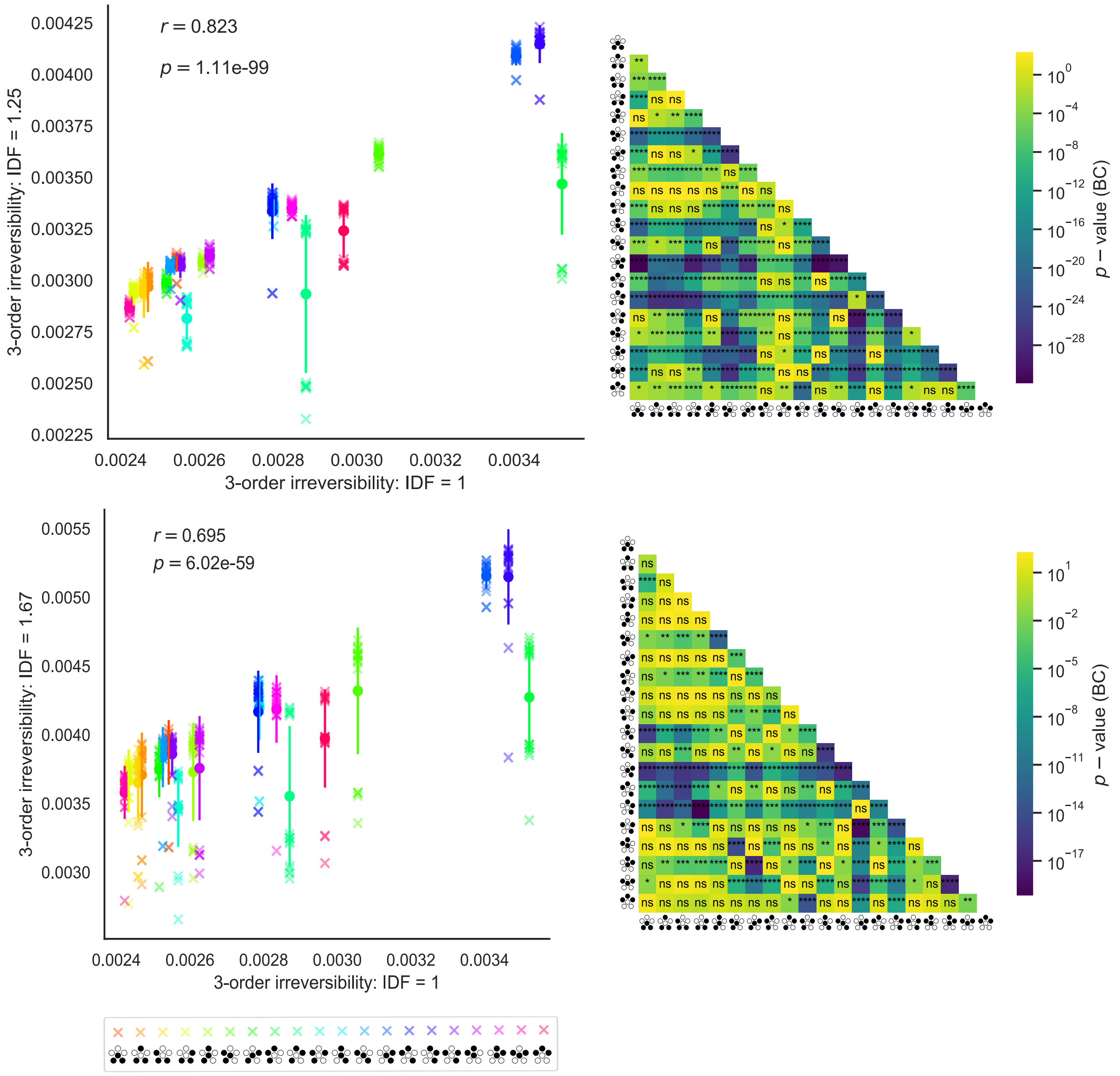

Fig. S7. Sub-sampling for estimation of finite-data errors at order 3. a) A comparison between the original results at IDF=1 and the results at 20 samples at IDF=1.25 (12/15 trials). We find that the results are highly correlated. b) Bonferroni-corrected paired $t-$tests show a range of significant pairwise comparisons. c) A comparison between the original results at IDF=1 and the results at 20 samples at IDF=1.67 (9/15 trials). We find that the results are highly correlated. d) Bonferroni-corrected paired $t-$tests show a range of significant pairwise comparisons.

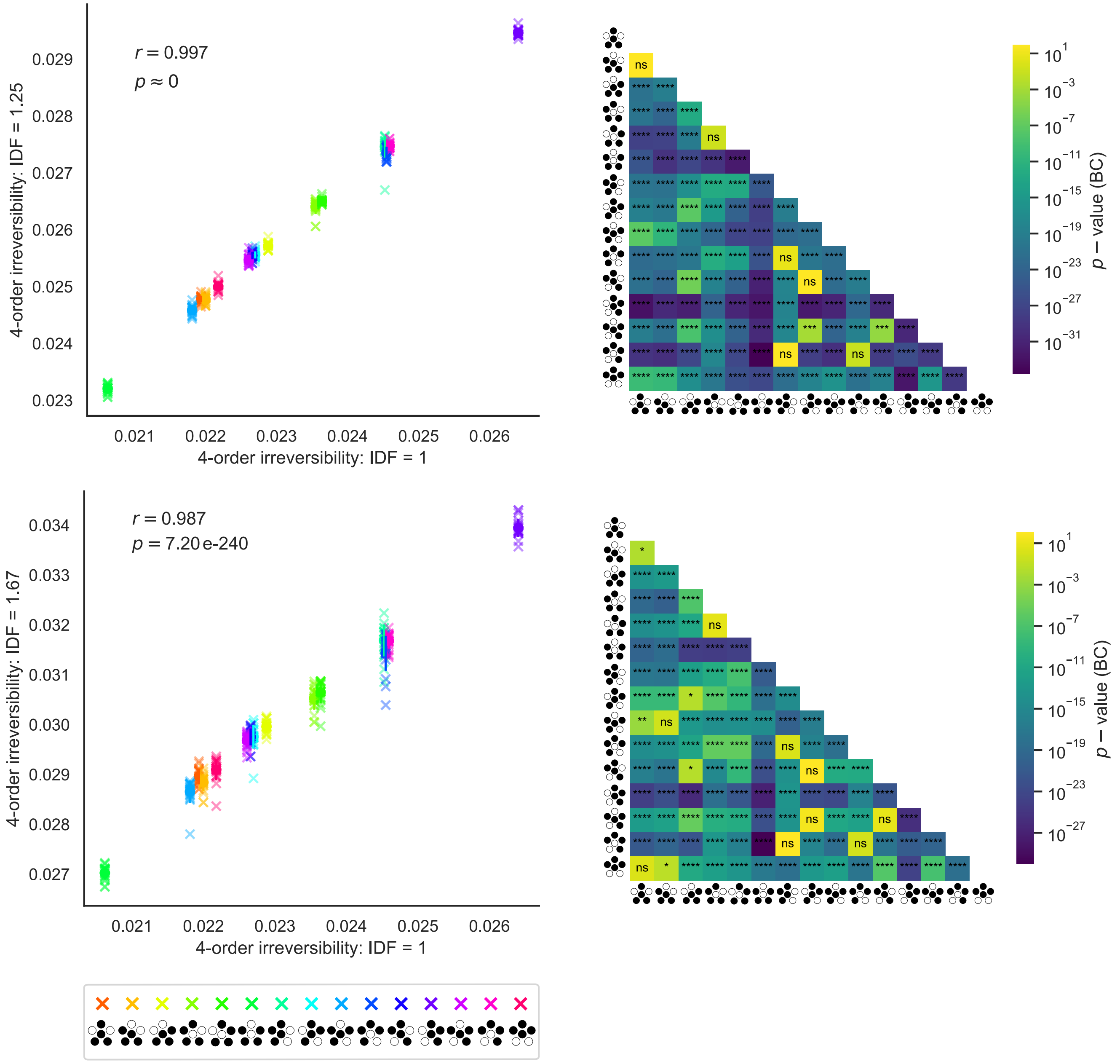

Fig. S8. Sub-sampling for estimation of finite-data errors at order 4. a) A comparison between the original results at IDF=1 and the results at 20 samples at IDF=1.25 (12/15 trials). We find that the results are highly correlated. b) Bonferroni-corrected paired $t-$tests show a range of significant pairwise comparisons. c) A comparison between the original results at IDF=1 and the results at 20 samples at IDF=1.67 (9/15 trials). We find that the results are highly correlated. d) Bonferroni-corrected paired $t-$tests show a range of significant pairwise comparisons.

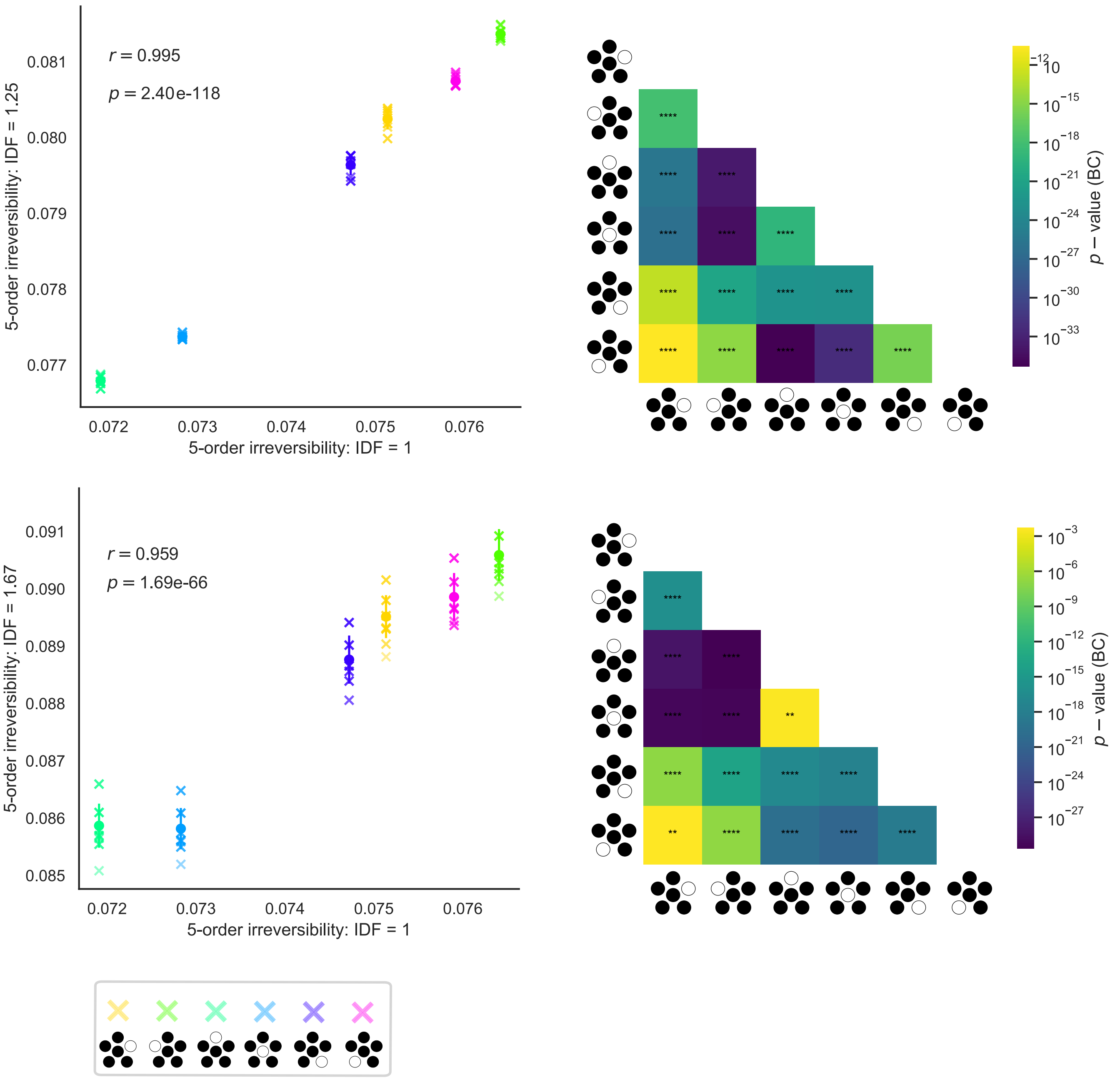

Fig. S9. Sub-sampling for estimation of finite-data errors at order 5. a) A comparison between the original results at IDF=1 and the results at 20 samples at IDF=1.25 (12/15 trials). We find that the results are highly correlated. b) Bonferroni-corrected paired $t-$tests show a range of significant pairwise comparisons. c) A comparison between the original results at IDF=1 and the results at 20 samples at IDF=1.67 (9/15 trials). We find that the results are highly correlated. d) Bonferroni-corrected paired $t-$tests show a range of significant pairwise comparisons.

multiplex factorises as follows,

$$P(d_1, ..., d_k) = P(d_1)P(d_2, ..., d_k). \tag{1}$$

Under the DiMViGI framework, we quantify the irreversibility of the tuplet as

$$\tilde{\varsigma}^{(x_1,...,x_k)} = \text{JSD}(P_{\text{in}}(d_1, ..., d_k), P_{\text{out}}(d_1, ..., d_k)), \tag{2}$$

$$= \sum_{d_1,...,d_k} P_{\text{in}}(d_1, ..., d_k) \log \frac{P_{\text{in}}(d_1, ..., d_k)}{P^*(d_1, ..., d_k)} + \sum_{d_1,...,d_k} P_{\text{out}}(d_1, ..., d_k) \log \frac{P_{\text{out}}(d_1, ..., d_k)}{P^*(d_1, ..., d_k)}, \tag{3}$$

where $P^* := \frac{1}{2}(P_{\text{in}} + P_{\text{out}})$. We focus first on the term concerning the in-degree distribution and use the independence of x_1 and the properties of logarithms to factorise and simplify this expression,

$$\sum_{d_1,...,d_k} P_{\text{in}}(d_1, ..., d_k) \log \frac{P_{\text{in}}(d_1, ..., d_k)}{P^*(d_1, ..., d_k)} = \sum_{d_1,...,d_k} P_{\text{in}}(d_1)P_{\text{in}}(d_2, ..., d_k) \log \frac{P_{\text{in}}(d_1)P_{\text{in}}(d_2, ..., d_k)}{P^*(d_1)P^*(d_2, ..., d_k)} \tag{4}$$

$$= \sum_{d_1,...,d_k} P_{\text{in}}(d_1)P_{\text{in}}(d_2, ..., d_k) \left(\log \frac{P_{\text{in}}(d_2, ..., d_k)}{P^*(d_2, ..., d_k)} + \log \frac{P_{\text{in}}(d_1)}{P^*(d_1)} \right) \tag{5}$$

$$= \sum_{d_2,...,d_k} P_{\text{in}}(d_2, ..., d_k) \log \frac{P_{\text{in}}(d_2, ..., d_k)}{P^*(d_2, ..., d_k)} + \sum_{d_1} P_{\text{in}}(d_1) \log \frac{P_{\text{in}}(d_1)}{P^*(d_1)}. \tag{6}$$

By symmetry the same is true for the term concerning the out-degree distribution. Substituting in the simplified expression, we get

$$\tilde{\varsigma}^{(x_1,...,x_k)} = \text{JSD}(P_{\text{in}}(d_2, ..., d_k), P_{\text{out}}(d_2, ..., d_k)) + \text{JSD}(P_{\text{in}}(d_1), P_{\text{out}}(d_1)), \tag{7}$$

$$= \tilde{\varsigma}^{(x_2,...,x_k)} + \tilde{\varsigma}^{(x_1)}. \tag{8}$$

This indicates that in this k-dimensional system that does not contain a genuine k-order interaction, the irreversibility of the $k-$tuple simply decomposes into the sum of non-independent tuples. By induction, for a $k-$tuple where all variables are independent, the irreversibility fully decomposes into the sum of the 1-order irreversibilities,

$$\tilde{\varsigma}^{(x_1,...,x_k)} = \sum_{i=1}^{k} \tilde{\varsigma}^{(x_i)}. \tag{9}$$

6. Unique contributions from higher order interactions

In our analysis, we have considered the irreversibility of multilevel interactions. However, for a given $k-$order interaction, we have measured the irreversibility of the combined $k-$tuple. This is in contrast with the 'unique' irreversibility that is contributed purely by the k-body interaction, discounting the $j-$body interactions for $j < k$ that are included within this $k-$tuple.

Within the theory of higher order interactions, this distinction represents the difference between a hyper-graphical structure and a simplicial complex (11). In the former, a k-body interaction does not comprise of lower-order components, whereas in the latter, every lower order relationship must exist to define a higher order one i.e. a $3-$order triangle relationship requires all the edges of the triangle to be included.

Within the lens of irreversibility, we note that the decomposition proposed by Lynn et al (6, 12) specifically considers the unique contributions to the global irreversibility. Alternatively, in the manuscript, we present a method that captures the irreversibility of path projected into the portion of state-space defined by a tuple,

$$\varsigma^{(x_1,...,x_k)} = \sum_{\Gamma^{(x_1,...,x_k)}} P(\Gamma^{(x_1,...,x_k)}) \log \frac{P(\Gamma^{(x_1,...,x_k)})}{P(\Gamma'^{(x_1,...,x_k)})}, \tag{10}$$

which is not equivalent to the unique contribution. In this section, we relate our framework more closely, but still not equivalently, to the decomposition in Ref. (12) by measuring the unique contribution of the k-body interaction to $\varsigma^{(x_1,...,x_k)}$. We do this by recursively subtracting the irreversibility of sub-tuples $\Omega \subset \{x_1, ..., x_k\}$, from the quantity $\varsigma^{(x_1,...,x_k)}$. In such a way we define the unique contribution to the $\varsigma^{(x_1,...,x_k)}$ of the $k-$body interaction $(x_1, ..., x_k)$ as,

$$\eta^{(x_1,...,x_k)} = \varsigma^{(x_1,...,x_k)} - \sum_{\Omega \subset \{x_1,...,x_k\}} \eta^{\Omega}, \tag{11}$$

which is calculable by noting that $\eta^{(x_i)} = \varsigma^{(x_i)}$. We are able to show that, using this framework, the results are highly correlated, indicating that higher order interactions dominate the irreversibility in these large-scale neural recordings. This stands in contrast with results obtained in spike-train data that indicate that, at the neuronal level, pairwise interactions dominate (6, 12).

We note that this approach captures the unique contributions of k-body interactions by considering the following

$$\varsigma^{(x_i)} = \eta^{(x_i)} \quad \forall i \in \{1, ..., N\}, \tag{12}$$

i.e. the combined and unique irreversibility at 1-order is equivalent. Next we note that for two independent variables, the irreversibility factorises, under the DiMViGI framework,

$$\varsigma^{(x_i,x_j)} = \eta^{(x_i)} + \eta^{(x_j)}. \tag{13}$$

For independent variables x_i, x_j, we expect $\eta^{(x_i,x_j)} = 0$. Therefore, it is natural to define,

$$\eta^{(x_i,x_j)} = \varsigma^{(x_i,x_j)} - \eta^{(x_i)} - \eta^{(x_j)}, \tag{14}$$

which is positive for correlated variables and vanishes for independent variables. By definition, it captures the irreversibility of the pairwise interaction, discounting the singleton dynamics. In this fashion, we can recursively calculate the unique contributions at k-order using the unique contributions at j-order for $1 \leq j < k$. Concretely, we have,

$$\eta^{(x_1,...,x_k)} = \varsigma^{(x_1,...,x_k)} - \sum_{\Omega \subset \{x_1,...,x_k\}} \eta^{\Omega}. \tag{15}$$

Figure S10 shows the contrast between the unique and combined irreversibilities for tuples at levels $k = 2, 3, 4$ at the cohort-level. We do not consider 1-order as the unique and combined values are equivalent. Furthermore, we cannot consider $k = 5$ as we employ degree-limiting (see Section 8) for computational efficiency at this level. As a result, we consciously underestimate the irreversibility at 5-order which leads to negative values when inputting these measurements into equation 15. Panel a) of Fig S10 shows a small level of contrast between the unique and combined pairwise dynamics. This indicates that the irreversibility of pairwise interactions dominates the irreversibility of singleton dynamics. Furthermore the general hierarchy is preserved. Panels b-c) show similar results with increasing levels of contrast. However, this increasing contrast is due to the combinatorics of higher order interactions. In particular, as we increase the level k, we are subtracting more terms when isolating the unique contribution. However, this difference is overstated, as panel d) shows that the correlation between unique and combined measurements is almost perfect. This indicates that at a given level k, the k-body interaction dominates the lower level interactions and contributes the most to the irreversibility. This result is both a consequence of the method, and the spatially-coarse, low-dimensional data under consideration. It further suggests that, whilst the DiMViGI framework can be used to compare irreversibility between levels, it is most useful for comparing tuples within a given level.

7. Validation using simulated data from the multivariate Ornstein-Uhlenbeck process

Next, we aim to validate our technique against simulated time-series. We choose the multivariate Ornstein-Uhlenbeck as this is one of the few models that has a known rate of entropy production (13). Furthermore, this model has been fit to neural recordings in the past in order to estimate the entropy production rate (14).

A. Multivariate Ornstein-Uhlenbeck process. The Orstein-Uhlenbeck process models the velocity of a particle in Brownian motion (15). In its generalised multivariate form, we consider N dimensions with coupled stochastic dynamics given by the equation,

$$dX(t) = -\boldsymbol{B}X(t) \;\; dt + \boldsymbol{\eta}(t), \tag{16}$$

where $X(t) \in \mathbb{R}^N$. The friction $-\boldsymbol{B} \in \mathbb{R}^{N\times N}$ is a stable matrix, meaning that every eigenvalue has strictly negative real part. The additive noise, $\boldsymbol{\eta}(t)$, is Gaussian and has covariance $\boldsymbol{D} \in \mathbb{R}^{N\times N}$,

$$\langle \boldsymbol{\eta}(t)\boldsymbol{\eta}^\top(t')\rangle = 2\boldsymbol{D}\delta(t - t'). \tag{17}$$

$\boldsymbol{D}$ is a symmetric, positive definite matrix, and so we can calculate $\boldsymbol{L}$, its Cholesky decomposition, where $\boldsymbol{L}$ satisfies $\boldsymbol{D} = \boldsymbol{L}\boldsymbol{L}^\top$ and $\boldsymbol{L}$ is lower triangular (13). As a result, we can write the system as a Langevin equation,

$$dX(t) = -\boldsymbol{B}X(t) \;\; dt + \boldsymbol{L}\, dW(t), \tag{18}$$

where $W(t)$ represents a N-dimensional Wiener process with independent components. The individual trajectories of a mOU are always reversible, yet at the macroscopic level, irreversibility can emerge. The macroscopic process is known to be reversible if $\boldsymbol{B}\boldsymbol{D}$ is symmetric i.e.,

$$\boldsymbol{B}\boldsymbol{D} = \boldsymbol{D}\boldsymbol{B}^\top. \tag{19}$$

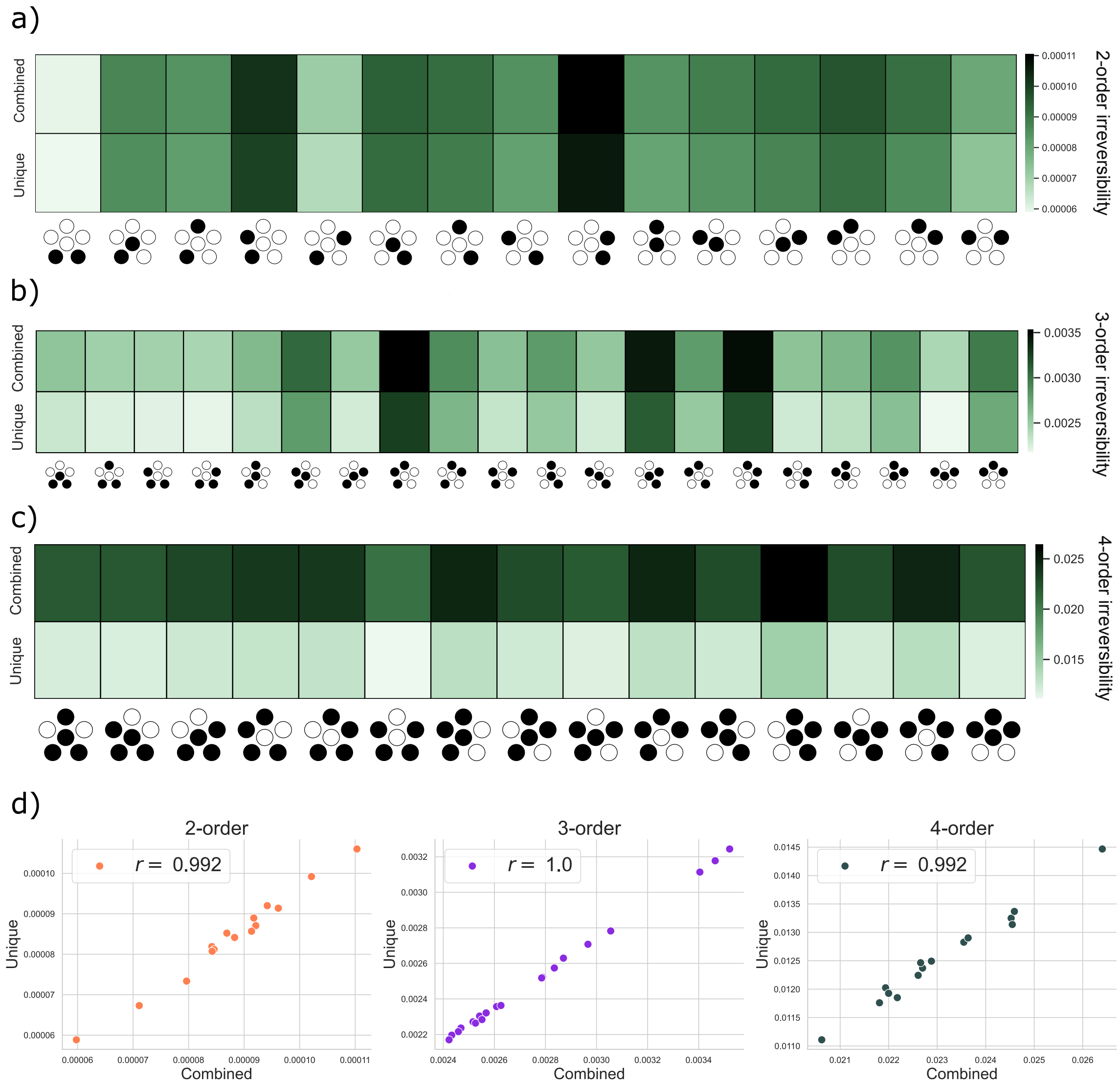

Fig. S10. Comparison of the combined k-order contributions to irreversibility against the unique contributions from each k-tuple for $k = 2, 3, 4$. a) $k = 2$. As the irreversibility of the pairwise interactions is much larger than the individual trajectories, the unique and combined irreversibilities are very similar. b) $k = 3$. Whilst there is some contrast between the unique and combined irreversibilities, the general hierarchy is preserved. c) $k = 4$. Again there is some contrast between the unique and combined irreversibilities with the general hierarchy being preserved. d) We show the almost perfect correlation between the unique and combined irreversibilities at each level. This indicates that the $k-$order interactions dominate the irreversibility at level k and suggest little difference when considering unique or combined irreversibilities.

Note that $\boldsymbol{D}$ is always symmetric, whereas, in general, $\boldsymbol{B}$ is not. Furthermore, the covariance, $\boldsymbol{S}$, of the stationary state can be defined implicitly in terms of $\boldsymbol{B}$ and $\boldsymbol{D}$ by the Lyapunov equation,

$$\boldsymbol{BS} + \boldsymbol{SB}^\top = 2\boldsymbol{D}. \qquad [20]$$

In the case that the process is reversible, we can use the criterion (19) to write $\boldsymbol{S}$ explicitly,

$$\boldsymbol{S} = \boldsymbol{B}^{-1}\boldsymbol{D}. \qquad [21]$$

In the case that the process is irreversible, obtaining an explicit form for $\boldsymbol{S}$ is not as simple. Instead, we parameterise the level of asymmetry using the Onsager matrix of kinetic coefficients and a matrix $\boldsymbol{Q}$, that represents the asymmetry,

$$\boldsymbol{L} = \boldsymbol{BS} = \boldsymbol{D} + \boldsymbol{Q}, \qquad [22]$$

$$\boldsymbol{L}^\top = \boldsymbol{SB}^\top = \boldsymbol{D} - \boldsymbol{Q}. \qquad [23]$$

As shown in (13), the entropy production rate for the multivariate Ornstein-Uhlenbeck process can be written in terms of the matrices $\boldsymbol{B}, \boldsymbol{D}$ and $\boldsymbol{Q}$. The rate of entropy production is given by,

$$\Phi = \mathrm{Tr}(\boldsymbol{B}^\top \boldsymbol{D}^{-1}\boldsymbol{B}) = -\,\mathrm{Tr}(\boldsymbol{D}^{-1}\boldsymbol{BQ}). \qquad [24]$$

Clearly, when the process is reversible, $\boldsymbol{Q} = 0$ and thus $\Phi = 0$. In general, the matrices $\boldsymbol{S}$ and $\boldsymbol{Q}$ cannot be determined in closed form and so Φ does not have a closed form expression. However, in the case $N = 2$ or in the presence of appropriate symmetries in the matrices $\boldsymbol{B}$ and $\boldsymbol{D}$, a closed form expression can be derived for Φ (13).

***A.1. The case* $N = 2$.** In the case $N = 2$, the Lyapunov equation (20) has a closed form solution, and therefore the entropy production rate can be explicitly expressed as a function of the entries of $\boldsymbol{B}$ and $\boldsymbol{D}$ (13).

Consider the matrices,

$$\boldsymbol{B} = \begin{pmatrix} a & b \\ c & d \end{pmatrix}; \qquad \boldsymbol{D} = \begin{pmatrix} u & w \\ w & v \end{pmatrix}. \qquad [25]$$

In this case, the rate of entropy production is given explicitly by the formula,

$$\Phi = \frac{(cu - bv + (d-a)w)^2}{(a+d)(uv - w^2)}. \qquad [26]$$

Clearly we have $\Phi = 0$ if and only if the reversibility criterion,

$$cu - bv + (d-a)w = 0, \qquad [27]$$

is satisfied (13).

A.2. Cyclic symmetry. Consider the situation where the variables live on a ring with N sites where the dynamics are invariant to translations of the ring. This results in the matrices $\boldsymbol{B}$ and $\boldsymbol{D}$ being circulant, i.e.

$$\boldsymbol{B} = \begin{pmatrix} b_0 & b_1 & \dots & b_{N-1} \\ b_{N-1} & b_0 & \dots & b_{N-2} \\ \vdots & \vdots & \dots & \vdots \\ b_1 & b_2 & \dots & b_0 \end{pmatrix}; \qquad \boldsymbol{D} = \begin{pmatrix} d_0 & d_1 & \dots & d_{N-1} \\ d_{N-1} & d_0 & \dots & d_{N-2} \\ \vdots & \vdots & \dots & \vdots \\ d_1 & d_2 & \dots & d_0 \end{pmatrix}. \qquad [28]$$

As $\boldsymbol{D}$ is assumed to be symmetric, this imposes the additional restriction that $d_{N-i} = d_i$. In this case, the rate of entropy production has a closed form expression,

$$\Phi = \sum_{k=0}^{N-1} \frac{(\Im(\tilde{b}_k))^2}{\Re(\tilde{b}_k)}, \qquad [29]$$

where $(\tilde{b}_0, ..., \tilde{b}_{N-1})$ is the discrete Fourier transform of the vector $(b_0, ..., b_{N-1})$, $\Im(\cdot)$ represents the imaginary part of a number and $\Re(\cdot)$ represents the real part (13). Recall that for a circulant matrix, the Fourier modes of $(b_0, ..., b_{N-1})$ coincide with the eigenvectors of $\boldsymbol{B}$.

B. Example processes validating the DiMViGI framework. Using the cases where we can calculate the explicit rate of entropy production, such as those detailed above, we can construct example processes and compare the measurements from our technique to the global rate of irreversibility. Figure S11 shows the results of these numerical experiments.

B.1. Example 1. We first consider Example 1, a 2-dimensional process with friction and noise given by,

$$\boldsymbol{B} = \begin{pmatrix} 4 & 1 \\ 2 & 1 \end{pmatrix}; \qquad \boldsymbol{D} = \begin{pmatrix} 1 + \frac{2}{2x+1} & 1 \\ 1 & 1 + \frac{2}{2x+1} \end{pmatrix}. \tag{30}$$

This gives rate of entropy production,

$$\Phi = \frac{4x^2}{5(2x+2)}, \tag{31}$$

which vanishes for $x = 0$, corresponding to a reversible process. Furthermore, as x increases from 0, the rate of entropy production grows linearly with x.

We numerically sample paths from this process for values $x = 0, 0.5, 1, ..., 10$ using an Euler-Maruyama scheme. We sample paths of length $T = 500$ with a time-step of $\Delta t = 0.01$ and keep only the last 2000 time-steps of the process, to avoid boundary effects.

As shown in Panel a) Fig. S11, we can see that the first order irreversibility captured by the DiMViGI techniques shows no correlation with the global rate of entropy production. This is because individual trajectories of the mOU are reversible. As a result, what is plotted is numerical error associated with finite trajectories which has no correlation with the parameters or Φ. On the other hand, the second order irreversibility of the pair (x_1, x_2) is capturing the global rate of entropy production as this interaction produces all the entropy in the system. As a result we can see the strong linear correlation between the 2-order irreversibility and Φ.

B.2. Example 2. We consider Example 2, a circulant 3-dimensional process with a strong triplet interaction,

$$\boldsymbol{B} = \begin{pmatrix} 1 & a & -a \\ -a & 1 & a \\ a & -a & 1 \end{pmatrix}; \qquad \boldsymbol{D} = \begin{pmatrix} 2 & 1 & 1 \\ 1 & 2 & 1 \\ 1 & 1 & 2 \end{pmatrix}. \tag{32}$$

This gives rate of entropy production,

$$\Phi = 6a^2. \tag{33}$$

For $a = 0.5, 1, ..., 8.5$, we sample paths with the same methods as before and estimate the irreversibility of the interactions in the system.

As shown in Panel b) of Fig. S11, the individual trajectories are again uncorrelated with the global rate as they are reversible. Both the pair and triplet dynamics are strongly correlated with the global rate of entropy production. Whilst we do not know how much each pair contributes to Φ, the circular symmetry of the process suggests the dynamics of pairs should be identical, which we see here.

B.3. Example 3. We consider Example 3, a circular 3-dimensional process with only pairwise drift interactions, given by,

$$\boldsymbol{B} = \begin{pmatrix} 1 & 0 & a \\ a & 1 & 0 \\ 0 & a & 1 \end{pmatrix}; \qquad \boldsymbol{D} = \begin{pmatrix} 2 & 1 & 1 \\ 1 & 2 & 1 \\ 1 & 1 & 2 \end{pmatrix} \tag{34}$$

which, again, gives rate of entropy production,

$$\Phi = 6a^2. \tag{35}$$

Paths are sampled for value $a = 0.1, 0.2, ..., 1.8$. Whilst each component is only coupled to itself and one other component directly in the drift matrix, it is coupled to the entire system via the noise matrix and indirectly via the dynamics of the other components. For example, even though x_2 does not appear in the drift term for x_1, they are correlated through shared noise and via x_3. For this reason, the difference between Example 2 and Example 3 is not extreme. As can be seen in Panel c) of Fig. S11, we get almost identical dynamics of the measure. Whilst, we aim to distinguish between Example 2 and Example 3, by restricting to pairwise or triplet dynamics, we note that the mOU is a linear system that can be decomposed into its pairwise interactions, meaning it cannot produce genuine higher order effects (11). However, we are restricted in this analysis to this model as the explicit entropy production rate is known.

B.4. Example 4. We consider Example 4 which is 4-dimensional and non-circulant. As a result, we no longer have the exact solution for the entropy production rate and must estimate this quantity numerically. Example 4 has drift and covariance,

$$\boldsymbol{B} = \begin{pmatrix} a^2 & a & 0 & 0 \\ a & a^2 & 0 & 0 \\ 0 & 0 & a^2 & a \\ 0 & 0 & a & a^2 \end{pmatrix}; \qquad \boldsymbol{D} = \begin{pmatrix} 2 & 1 & 1 & 1 \\ 1 & 2 & 1 & 1 \\ 1 & 1 & 2 & 1 \\ 1 & 1 & 1 & 2 \end{pmatrix}. \tag{36}$$

This system is interacting as a 4-dimensional system as it is coupled through the noise dynamics. However, in the drift term we have two subsystems where (x_1, x_2) interact strongly as do (x_3, x_4), but these pairs are drift-wise disjoint. In order to numerically estimate the entropy production rate, we estimate the covariance matrix from the sampled paths,

$$\boldsymbol{S} = \langle X, X^\top \rangle. \tag{37}$$

Next, we can calculate the asymmetric part of the Onsager matrix,

$$\boldsymbol{Q} = \frac{1}{2}\left(\boldsymbol{BS} - \boldsymbol{SB}^\top\right), \tag{38}$$

which can be used to calculate the entropy production rate,

$$\Phi = -\operatorname{Tr}(\boldsymbol{D}^{-1}\boldsymbol{BQ}). \tag{39}$$

We sample paths for values $a = 2.5, 2.7, ..., 4.9$, but we do not know how Φ scales with a. Panel d) of Fig. S11 is harder to interpret than for the previous examples, as the numerical approach produces greater variance in the plot. However, we plot least-square regression lines for each tuple. At 1-order, the lines are almost flat, as expected as there should be no correlation between the reversible individual trajectories and the underlying rate of entropy production. At 2-order, we have the most important result, which is that whilst the reversibility of all pairs scales with entropy production, the strongly interacting pairs (x_1, x_2) and (x_3, x_4), the upper two lines, are more irreversible. At 3-order, all the interactions produce almost identical amounts of irreversibility, which is to be expected as each triplet contains a strongly interacting pair and one component from the other pair, leading to a symmetry in the dynamics. Finally, the irreversibly of the quadruplet, the entire system, scales linearly with the underlying entropy production rate.

We note that the mOU is not a truly higher-order system as it is linear and the interactions can be seen as pairwise, but we are restricted to this model as it has a known rate of entropy production and producing continuous dynamics. Other techniques have validated their techniques on chaotic processes (16) or symbolic dynamics i.e. Ising model (3). However, deterministic chaos and thermodynamic irreversibility are not equivalent. Furthermore, these processes do not allow one to scale the number of variables, nor the level of irreversibility, arbitrarily. On the other hand, by varying the thermodynamic temperature, one can vary the irreversibility of the Ising model, but the visibility graph is designed to capture correlations in continuous rather than binary series yielding this model unsuitable. For this reason, we opt exclusively for the mOU as studied here.

8. Varying the maximum degree in the support of the degree distributions

The DiMViGI framework projects the high-dimensional, continuous state-space of the multivariate time-series into a discrete and low-dimensional representation using the visibility graph, thus reducing the computational cost of calculating information-theoretic quantities (17, 18). However, the combinatorial complexity of considering every possible tuple in a system can be restrictive. Furthermore, estimating high-dimensional degree distributions can also be computationally demanding in terms of computer memory. A simple method for improving the memory efficiency of the DiMViGI framework is to cap the maximum degree in the support of the degree distribution. The degree distribution of the visibility graph typically decays exponentially as the degree increases (17, 19, 20). As a result, when limiting the degree, we are removing minimal information. Moreover, a k-dimensional distribution with maximum degree $d_{\max}$ contains $d_{\max}^k$ entries. Therefore, degree-limiting has an exponential reduction in the memory usage of the DiMViGI implementation. In our analysis presented in the main manuscript, we employed degree limiting in the case of $k = 5$, where we enforced $d_{\max} = 75$. In this section, we present a systematic analysis of the effect of degree limiting for each tuple at each level. We implement this limiting by enforcing that if a node has a degree greater than $\tilde{d}_{\max}$, we set its degree to $\tilde{d}_{\max}$ in the distribution.

First, we note that, in practice, the restriction causes us to underestimate the irreversibility of the tuple. However, this is not mathematically guaranteed. For a tuple, $(x_1, ..., x_k)$, we denote the irreversibility with full support to be $\tilde{\varsigma}^{(x_1,...,x_k)}$ and the irreversibility with limited support to be $\hat{\varsigma}^{(x_1,...,x_k)}$. Therefore, the difference is,

$$\Delta = \tilde{\varsigma}^{(x_1,...,x_k)} - \hat{\varsigma}^{(x_1,...,x_k)}. \tag{40}$$

The sign of Δ reflects whether we are over or underestimating the irreversibility using the limited support. We recall the definition of JSD between distributions P and Q,

$$J(P|Q) = \frac{1}{2}D(P|M) + \frac{1}{2}D(Q|M), \tag{41}$$

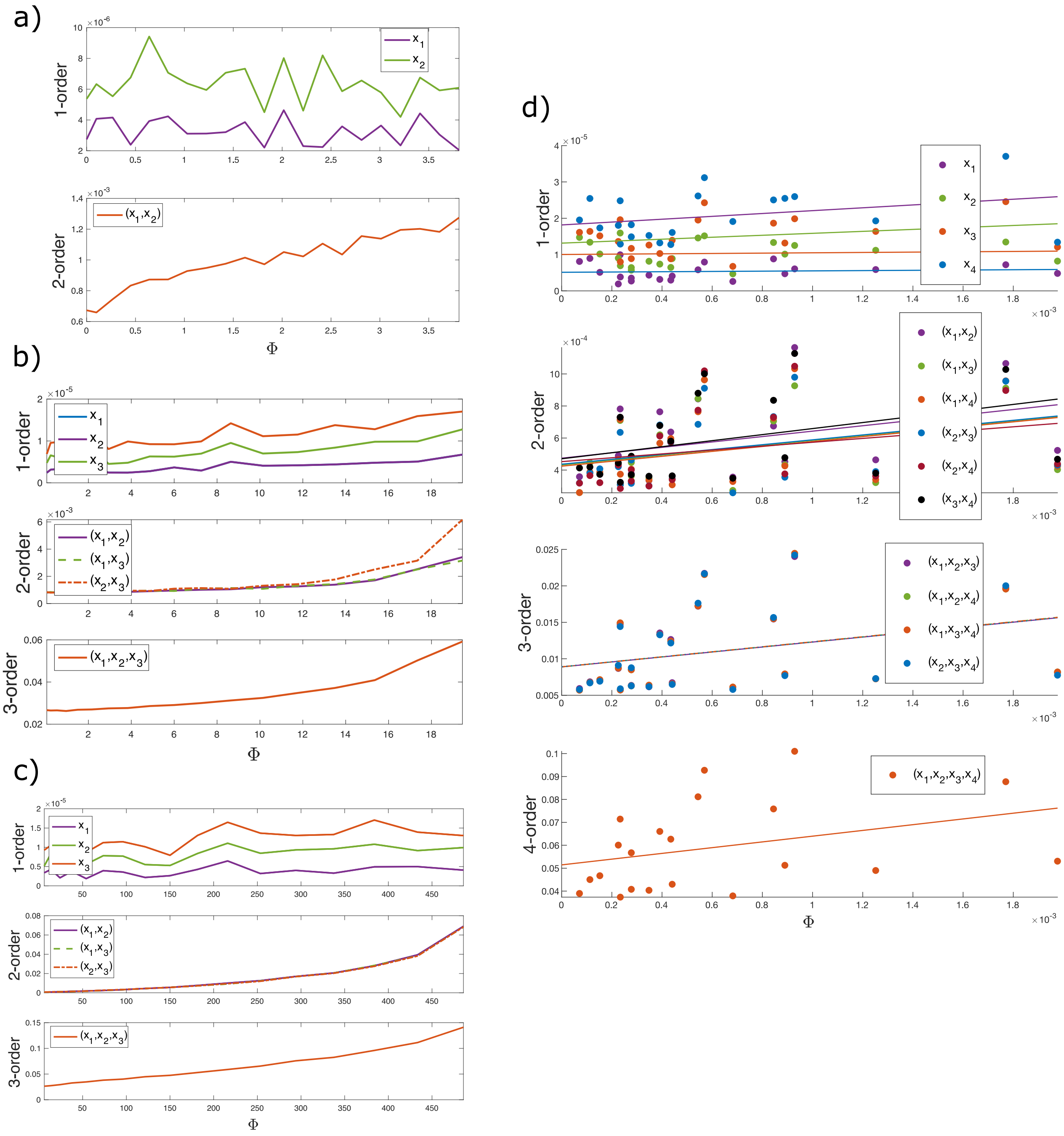

Fig. S11. Validation of the DiMViGI framework using simulated data from the mOU. a) Example 1 - a 2-dimensional process. The pairwise irreversibility scales with the global rate Φ, whilst the individual variables do not. b) Example 2 - a 3 dimensional process with drift-disjoint pairs. The pairs and triplet irreversibilities scale with the global rate Φ whilst the individual trajectories do not. c) Example 3 - a 3 dimensional process with 3 way interactions in the drift and noise. The pairs and triplet irreversibilities scale with the global rate Φ whilst the individual trajectories do not. d) Example 4 - a 4 dimensional process with 2 strongly interacting pairs. The global rate Φ is estimated numerically producing variance in the plot so we plot least-square regressions. The pairs and triplets and quadruplet irreversibilities scale with the global rate Φ whilst the individual trajectories do not. Notably, the strongly interacting pairs (x_1, x_2) and (x_3, x_4) have a higher level of irreversibilities than the other pairs.

where $M = \frac{1}{2}(P+Q)$ is an averaged distribution and $D(\cdot)$ represents the KLD, given by,

$$D(P|Q) = \sum_{x\in\mathcal{X}} P(x)\log\frac{P(x)}{Q(x)}. \tag{42}$$

Therefore,

$$\Delta = \left(\frac{1}{2}\sum_{d_1=\tilde{d}_{\max}+1,\dots,d_k=\tilde{d}_{\max}+1}^{d_{\max}} P_{\text{in}}\log\frac{P_{\text{in}}}{P^*} + P_{\text{out}}\log\frac{P_{\text{out}}}{P^*}\right) \tag{43}$$

$$+\frac{1}{2}\left(\sum_{d_i=\tilde{d}_{\max}\text{ for some } i} P_{\text{in}}\log\frac{P_{\text{in}}}{P^*} + P_{\text{out}}\log\frac{P_{\text{out}}}{P^*} - \tilde{P}_{\text{in}}\log\frac{\tilde{P}_{\text{in}}}{\tilde{P}^*} - \tilde{P}_{\text{out}}\log\frac{\tilde{P}_{\text{out}}}{\tilde{P}^*}\right), \tag{44}$$

where $P_{\text{in}}, P_{\text{out}}$ are the in- and out-degree distributions with the full support; $\tilde{P}_{\text{in}}, \tilde{P}_{\text{out}}$ are the in- and out-degree distributions with the limited support and $P^*, \tilde{P}^*$ are the averaged in-out distributions. In other words, the limited and full degree distributions overlap for all degrees $d < \tilde{d}_{\max}$ and therefore cancel when we take the difference. By truncating the distribution we are neglecting a number of positive terms from the full support. However, we must also consider that these edges have not been simply deleted, but are now included in overestimating the probability that a node has degree $\tilde{d}_{\max}$, hence P and $\tilde{P}$ differ when a node has degree $\tilde{d}_{\max}$ in some layer. We can rewrite Δ as,

$$\Delta = A - \frac{1}{2}\left(\sum_{d_i=\tilde{d}_{\max}\text{ for some } i} \tilde{P}_{\text{in}}\log\frac{\tilde{P}_{\text{in}}}{\tilde{P}^*} + \tilde{P}_{\text{out}}\log\frac{\tilde{P}_{\text{out}}}{\tilde{P}^*} - P_{\text{in}}\log\frac{P_{\text{in}}}{P^*} - P_{\text{out}}\log\frac{P_{\text{out}}}{P^*}\right), \tag{45}$$

where $A \geq 0$. Therefore, if the subtracted sum is less than A, we will underestimate the irreversibility, but if it greater than A we will overestimate the irreversibility. As shown in Figure S12, in practice, we consistently underestimate the irreversibility by limiting the degree, indicating that A is much larger than the subtracted term.

Figure S12 shows a systematic variation of the maximum degree. We perform the analysis with $\tilde{d}_{\max} = \alpha d_{\max}$ and $\alpha \in [0,1]$, where $d_{\max}$ is the maximum degree of the full multiplex visibility graph (MVG) from the data for $k = 1, ..., 4$ and $d_{\max} = 75$ for $k = 5$. We vary $\alpha = 0, 0.1, ..., 0.9, 1$ and calculate the irreversibility of each tuple using the DiMViGI framework but with restricted degree distribution. In addition, we also show, for each value of α, the Pearson correlation coefficient, r, and Spearman's rank, ρ, between the limited and full support values at each level. Panels a-e) show the effect on the irreversibilities of k-tuples with $k = 1, ..., 5$ respectively and panel f) recalls the schematic representation of the regions of interest. For each level, the irreversibility monotonically increases as we increase α, confirming that degree-limiting underestimates the irreversibility. For lower orders (1-2), we see that the increase is linear. In particular, for the pairwise results, to get a strong correlation with the full support irreverisibility, one needs to use a large proportion of the $d_{\max}$. On the other hand, for the higher orders (3-5), the increase is sigmoidal. Panels c-e) indicate that even limiting to half of the maximum degree is sufficient for an almost perfect correlation with the original results. For order 4-5, we see that this also captures approximately 90% of the irreversibility. With an exponentially smaller distribution, one can capture almost equivalent information. This analysis indicates that degree limiting is a very practical and useful tool to maximise the memory efficiency of the DiMViGI framework at higher orders.

9. Formulations of the entropy production rate for Markovian and non-Markovian systems

For certain systems in a stationary state, the entropy production rate (EPR) can be explicitly formulated.

In the case of discrete time, discrete space, Markovian dynamics, the entropy production rate simplifies to,

$$\sigma = \sum_{i,j} P_{ij}\log\frac{P_{ij}}{P_{ji}}, \tag{46}$$

where P_{ij} is the join transition probability, $P(x_{t+1} = j, x_t = i)$ (3).

For $l-$order Markovian dynamics, the rate of entropy production is given by,

$$\sigma = \frac{1}{l}\sum_{x_1,\dots x_{l+1}} P_{x_1,\dots,x_{l+1}}\log\frac{P_{x_1,\dots,x_{l+1}}}{P_{x_{l+1},\dots,x_1}}, \tag{47}$$

where $P_{x_1,\dots,x_{l+1}}$ is the probability of observing the exact sequence of states $x_1, ..., x_{l+1}$ (3).

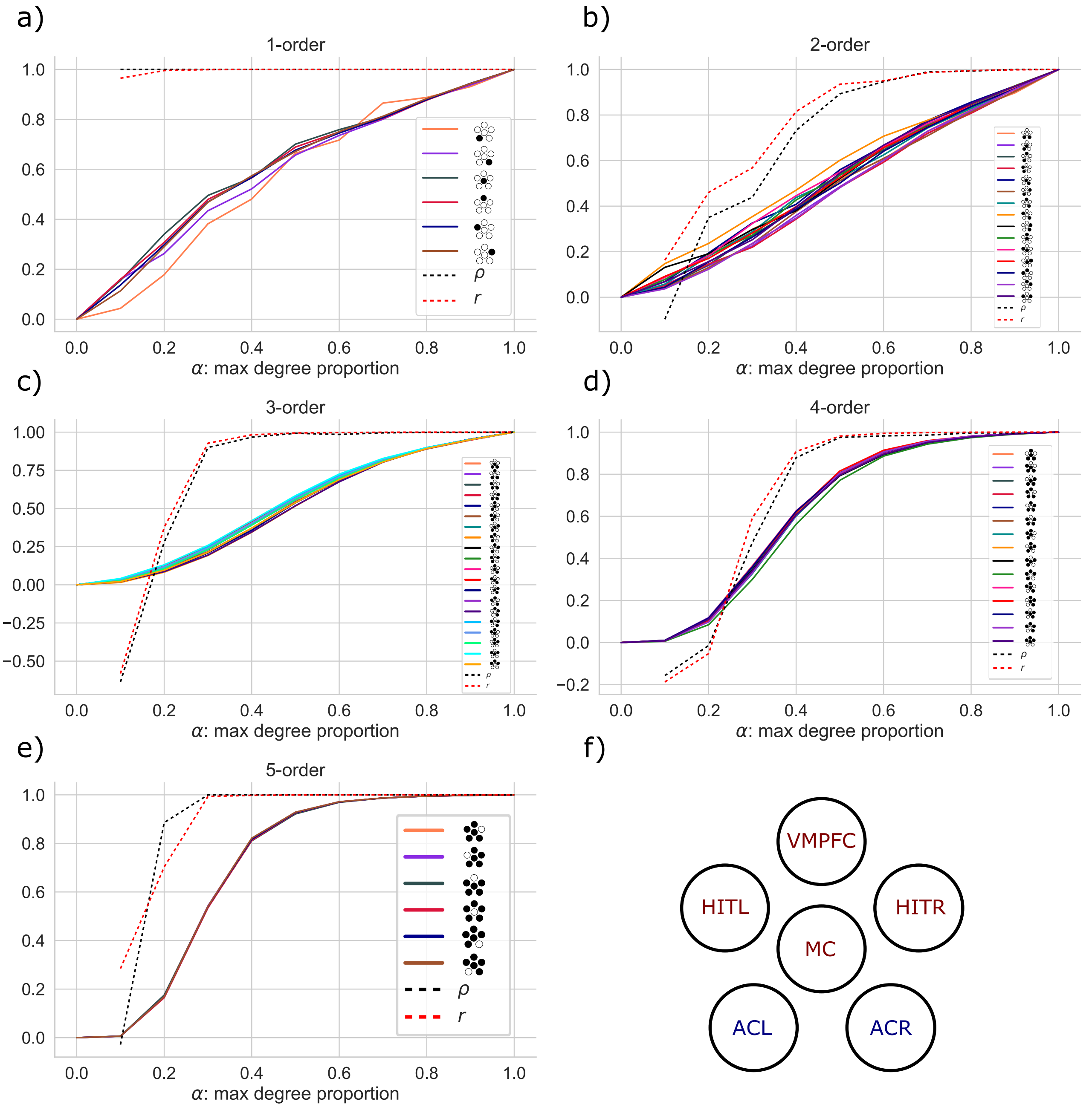

Fig. S12. Systematic analysis of the effect of degree-limiting on the irreversibility of each tuple. Panels a-e) show the proportion of irreversibility captured with degree limited to $\tilde{d}_{\max} = \alpha d_{\max}$ for $\alpha \in [0, 1]$. Using a limited degree underestimates the irreversibility of the tuple. In addition a-e) show the correlation between the limited degree results at each level, and the full degree results. For higher orders, degree limiting is shown to lose minimal information, both in terms of the correlations between tuples and absolute values. This indicates that is is a valuable technique for maximising the efficiency of the DiMViGI framework. Panel f) recalls the schematic representation for the tuples in the legends.

For a general system with discrete states in discrete time, the rate of entropy production of a single trajectory is given by,

$$\sigma = \lim_{t\to\infty} \frac{1}{t} \sum_{x_1,\ldots x_{t+1}} P_{x_1,\ldots,x_{t+1}} \log \frac{P_{x_1,\ldots,x_{t+1}}}{P_{x_{t+1},\ldots,x_1}}, \tag{48}$$

where $P_{x_1,\ldots,x_{t+1}}$ is the probability of observing the exact sequence of states $x_1, \ldots, x_{t+1}$ (3).

For continuous time Markovian dynamics, the time-dependent rate of entropy production is given by,

$$\sigma(t) = \frac{1}{2} \sum_{i,j} [p_i(t)w_{ij} - p_j(t)w_{ji}] \log \frac{p_i(t)w_{ij}}{p_j(t)w_{ji}}, \tag{49}$$

where $p_i(t)$ is the instantaneous probability distribution and w_{ij} are the transition rates (21).

For Markovian dynamics in continuous space and time, given by the Langevin equation,

$$\dot{\mathbf{x}} = A(\mathbf{x}, t) + B(\mathbf{x}, t) \cdot \eta(t), \tag{50}$$

we can write an equivalent Fokker-Planck equation,

$$\partial_t p(\mathbf{x}, t) = -\nabla \mathbf{j}(\mathbf{x}, t) \tag{51}$$

$$\mathbf{j}_i(\mathbf{x}, t) = A_i(\mathbf{x}, t)p(\mathbf{x}, t) - \sum_j \nabla_j (D_{ij}(\mathbf{x}, t)p(\mathbf{x}, t)), \tag{52}$$

where,

$$D(\mathbf{x}, t) = \frac{1}{2} B(\mathbf{x}, t)B(\mathbf{x}, t)^\top. \tag{53}$$

Following (22), the time-dependent entropy production rate is given by,

$$\sigma(t) = \int \mathbf{x}\; F(\mathbf{x}, \mathbf{t})\mathbf{j}(\mathbf{x}, t), \tag{54}$$

where,

$$F(\mathbf{x}, t) = \frac{\mathbf{j}^\top(\mathbf{x}, t)D(\mathbf{x}, t)^{-1}}{p(\mathbf{x}, t)}. \tag{55}$$

10. Experimental paradigm and MEG recordings

In this section, we provide additional information about the experimental paradigm, acquisition and pre-processing of the MEG recordings.

A. Experimental paradigm. We employed an old/new paradigm auditory recognition task (23–28). Participants listened the first four bars of the right-hand part of Johann Sebastian Bach's Prelude No. 2 in C Minor, BWV 847, twice and were asked to memorise it to the best of their ability. Next, participants listened to 135 five-tone musical sequences, corresponding to 27 trials in 5 experimental conditions, of 1750 ms each and were requested to indicate if the sequence belonged to the original music or was a variation. The experimental conditions corresponded to systematic variations on the position of the first varied tone in the sequence. For a detailed description and analysis of the different experimental conditions, see Bonetti et al (23). We consider one experimental condition, where participants recognised the original, previously memorised sequences.

B. Participants. The participant cohort consisted of 83 healthy volunteers made up of 33 males and 50 females with ages in the range 18 to 63 and a mean age of 28.76 ± 8.06. The 51 participants included in this analysis included 22 males and 29 females with ages in the range 18 to 63 and a mean age of 27.57 ± 7.13. Participants were recruited in Denmark, came from Western countries, reported normal hearing and gave informed consent before the experiment. Healthy aging can result in changes to auditory processing (29). Whilst the age-range considered in this study is large, Fig. S13 shows that the vast majority of participants are young. As a result, age related factors are deemed not to represent an issue with regards to our analyses.

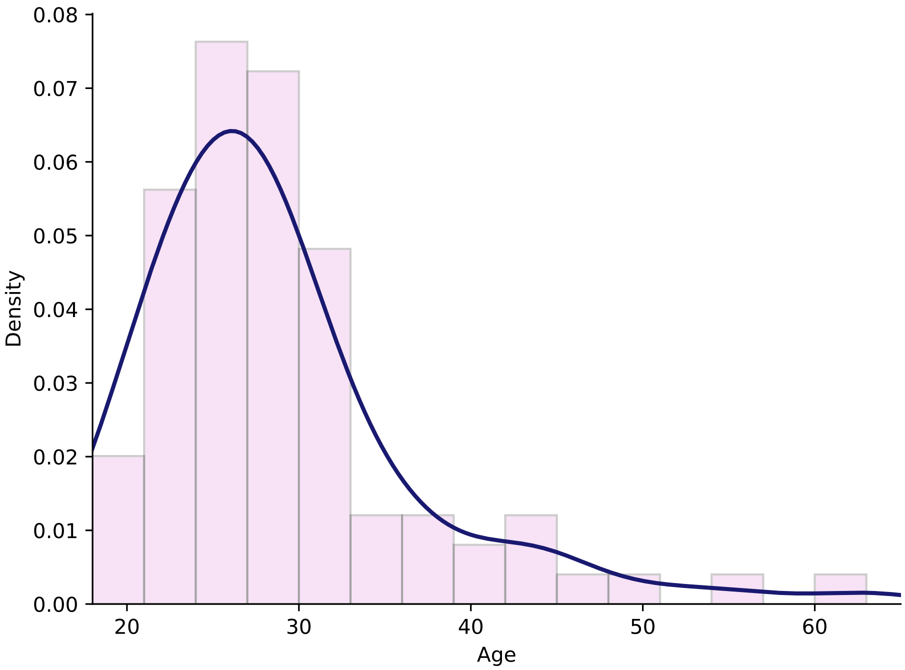

Fig. S13. Age-distribution of participants. The age distribution of participants shows that, despite a large range, the vast majority of participants were young, thus age-related changes are not significant for this study.

C. Data acquisition. The MEG recordings were taken in a magnetically shielded room at Aarhus Univeristy Hospital (AUH), Aarhus, Denmark on an Elekta Neuromag TRIUX MEG scanner with 306 channels (Elekta Neuromag, Helsinki, Finland). The sampling rate was 1000 Hz with analogue filtering of 0.1-330 Hz. Before taking the recordings, we registered the head shape of participants and the position of four Head Position Indicator (HPI) coils with respect to three anatomical landmarks using a 3D digitiser (Polhemus Fastrak, Colchester, VT, USA). We used this recording to co-register MRI scans with the MEG recordings. During the MEG recordings, the HPI coils continuously registered the localisation of the participant's head which was then used for movement correction. Furthermore, heartbeats and eye-blinks were recorded with two sets of bipolar electrodes which were then used, further along the pre-processing pipeline, to remove artefacts from the MEG recordings. The MRI scans were taken on a CE-approved 3T Siemens MRI-scanner at AUH. The MRI data consisted of structural T1 (mprage with fat saturation) with a spatial resolution of 1.0 x 1.0 x 1.0 mm and the following sequence parameters: echo time (TE) = 2.61 ms, repetition time (TR) = 2300 ms, reconstructed matrix size = 256 x 256, echo spacing = 7.6 ms, bandwidth = 290 Hz/Px. The MRI and MEG recordings were acquired on two separate days.

D. MEG data pre-processing. Firstly, the raw MEG sensor data (204 planar gradiometers and 102 magnetometers) was pre-processed using MaxFilter to attenuate external interferences (30). Next, signal space separation was applied with MaxFilter parameters: spatiotemporal signal space separation [SSS], down-sample from 1000Hz to 250Hz, movement compensation using cHPI coils [default step size: 10 ms], correlation limit between inner and outer subspaces used to reject overlapping intersecting inner/outer signals during spatiotemporal SSS: 0.98). Then the data was converted into Statistical Parametric Mapping (SPM) formatting and further pre-processed in MATLAB (MathWorks, Natick, MA, USA) using in-house-built codes (LBPD, available at https://github.com/leonardob92/LBPD-1.0.git) and the Oxford Centre for Human Brain Activity (OHBA) Software Library (OSL) (available at https://ohba-analysis.github.io/osl-docs/) (31). OSL is freely available software that builds on the Fieldtrip (32), FSL (33), and SPM (34) toolboxes. Next the continuous MEG data was visually inspected and large artefacts were removed. This removal discarded less than 0.1% of the data. Independent component analyses (ICA) were used to removed artefacts stemming from heart-beats and eye-blinks (35). Firstly, the original signal was decomposed into independent components. Next, we isolated and discarded the components that picked up activity from eye-blinks and heartbeats. Then the signal was rebuilt using the remaining components. Lastly, the signal was epoched into 135 trials, 27 trials in 5 experimental conditions, and the mean baseline signal, obtained from the post-stimulus brain signal, was removed. Each resulting trial lasted 4500 ms, made up of 4400 ms plus 100 ms of baseline time.

E. Source reconstruction. Whilst MEG recordings have excellent temporal resolution when compared to other imaging modalities, one must employ source-reconstruction to spatially locate activity in the brain. We employed the beam-forming algorithm (36–38) implemented in both in-house codes and OSL, SPM and FieldTrip.

In the following, we give a thorough description of the inverse model employed in the beam-forming algorithm. The algorithm is made up of two steps: (1) designing a forward model, (2) computing the inverse solution.

The forward model is a theoretical model that considers each brain source as a voxel/active source. The model describes how the strength of each dipole would be reflected onto each of the MEG sensors. We employed magnetometer channels and an 8-mm grid, which returned 3559 dipole locations (voxels) within the whole brain. We co-registered the individual, structural T1 data with the fiducial points and then computed a forward model by adopting the 'Single Shelf' method (39). This outputs the so-called 'leadfield' model which is an $S \times M$ matrix, L, where S is the number of sources and M is the number of MEG channels. In three cases, the structural T1 was not available and so we performed the leadfield computation with the 'MNI152-T1 with 8-mm spatial resolution' template.

Next, we used the beam-forming algorithm to compute the inverse solution. By sequentially applying a set of weights to the source locations, the algorithm can isolate the contribution of each source to the activity recorded by each MEG channel at each time-point of the recording. We summarise the beamforming algorithm in the following steps.

Firstly, the data recorded by the MEG sensors B at time t is described by the equation,

$$B_t = LQ_t + \nu \quad [56]$$

where L is the leadfield model, Q is the 'dipole matrix' which carries the activity of each dipole over time and ν is noise (37). The aim is to compute Q by solving the inverse problem. In the beam-forming algorithm, weights are computed and then applied to B_t i.e. for a single dipole, q, we have,

$$q_t = W^\top B_t. \quad [57]$$

Beam-forming computes weights, W_n, for each brain source n using the covariance matrix of the MEG sensors, C, calculated on the continuous signal with all trials concatenated, in the following fashion,

$$W_n = (L_n^\top C^{-1} L_n)^{-1} L_n^\top C^{-1}. \quad [58]$$

Following Nolte (39), the computation of the leadfield method was performed for three orientations of each brain source. Using singular value decomposition (SVD), the three orientations were reduced to one,

$$L = \mathrm{SVD}(\tilde{L}^{\top} C^{-1} \tilde{L})^{-1}, \qquad [59]$$

a common technique for simplifying beam-forming output (40, 41). Here, $\tilde{L}$ represents the leadfield model with three orientations. Lastly, the obtained weights were applied to each brain source at each time-point and normalised according to Luckhoo et al (41). In addition to individual trials, the weights were applied to averaged neural activity over all trials. The procedure returned a time-series for each of the 3559 brain sources for each trial, referred to as the 'neural activity index'. The sign ambiguity of the evoked responses time series was adjusted for each brain source using its sign in correspondence with the N100 response to the first tone of the auditory sequences (see Refs. (23, 25–27)).

Finally, the 3559 voxels obtained through source reconstruction were reduced to six functional brain parcels (or regions of interest (ROIs)) that roughly correspond to auditory cortices in the left and right hemispheres (ACL, ACR); the hippocampal and inferior temporal cortices in the left and right hemispheres (HITL, HITR) and two medial regions, the bilateral medial cingulate gyrus (MC) and the bilateral ventro-medial prefrontal cortex (VMPFC). Our functional ROIs were defined in a prior study using a larger dataset, from which we analyse a subset, to explore the event-related neural responses underlying auditory recognition (23). The full dataset in the original study included 83 participants across 5 experimental conditions. These conditions were highly similar, with participants listening to a memorized piece of music, either with no variation (Condition 1) or with variations in tone (Conditions 2-5). For the current study, we focus on a subset of this dataset, comprising 51 participants, and analyse only Condition 1. The decision to focus on a single condition was based on the nature of our analysis, which emphasises comparisons between tuples of ROIs of different orders rather than condition-based comparisons. Moreover, the prior study (23) demonstrated low variability within the full sample of 83 participants, with minimal standard errors across conditions. This suggests that the functional ROIs defined in this larger sample are well-representative of our subsample of 51 participants. Additionally, since the five experimental conditions elicited very similar brain activation patterns, as shown in (23), focusing on Condition 1 is both justified and appropriate for the current analysis. In Ref. (23), an extensive description and validation of these functionally derived ROIs is provided. Moreover, the study showed the consistency of the results when comparing these ROIs with ROIs taken from the well-known Automated Anatomical Labelling (AAL) parcellation and encompassing bilateral Heschl's gyri, bilateral hippocampi and medial and anterior cingulate cortex. Thus, in order to integrate our results with previous and future studies, we opt to use the same functional ROIs, which have been validated in detail in Ref. (23). The data was analysed at a temporal resolution of 4 ms so the resulting multivariate time-series was of dimension 6×1026 (variables $\times$ time-points).

11. Signal quality and signal to noise ratio in MEG recordings

In this experimental paradigm, 135 trials were performed per participant for 83 participants, corresponding to 27 trials and 5 experimental conditions (23). In this study, we focused on a single experimental condition. Furthermore, as analysis is restricted to participants who correctly indicated that the piece of music had not been altered, this means that each participant had a unique number of successful, usable trials up to a maximum of 27. We considered data from the 51 participants who had at least 15 successful, usable trials out of the total 83 participants. For those participants who had more than 15 successful trials, we then randomly sampled 15 trials in order to have an equal contribution from each participant, allowing for the participant level analysis presented in Sec. 2. Recommendations for trial counts in an MEG paradigm differ by task, but 40-100 trials is often considered a sensible range (42). In order to validate our choice to use 15 trials, even for participants who had a larger number of available trials, we consider the participant-trial-averaged signal shown in panel a) of Figure S14. The 6 panels show the average signal of each region, indicated by the schematic representation. The blue lines represent the data used in this study, taken by first averaging over the 15 trials per participant and then averaging over all participants. The shading represents the standard error of each participant compared to the mean signal. The yellow dashed lines represent the mean signal using the total available data with a custom number of trials per participant, with a minimum of 15 and a maximum of 27. This figure highlights the strong neural response associated with this task-paradigm, as found in previous studies (23–28). Further, the strong similarity between the yellow and blue signals indicates that opting for a smaller number of trials in order to have the same contribution from each participant, does not result in a decrease in signal quality or signal to noise ratio.

In panel b) of Figure S14, we extend this analysis by focusing on the pre-stimulus time, where the red line indicates the stimulus-time. Panel b) shows that the recordings contain a 'clean' baseline level of activity with small fluctuations around 0, followed by a strong response after the stimulus.

Each recording contains an epoch of 1026 time-points representing 0.1 seconds of baseline time, followed by a 4 second of active period. The full epoch was considered in the analysis. For further discussion of the paradigm and the associated data see Ref. (23).

12. Epoch comparison

In both the main manuscript and the SI, in order to use the maximum amount of data, we analyse the full epoch of 4.1 seconds, made up of 0.1 seconds of pre-stimulus baseline time and the following 4 seconds of post-stimulus activity. Alternative choices

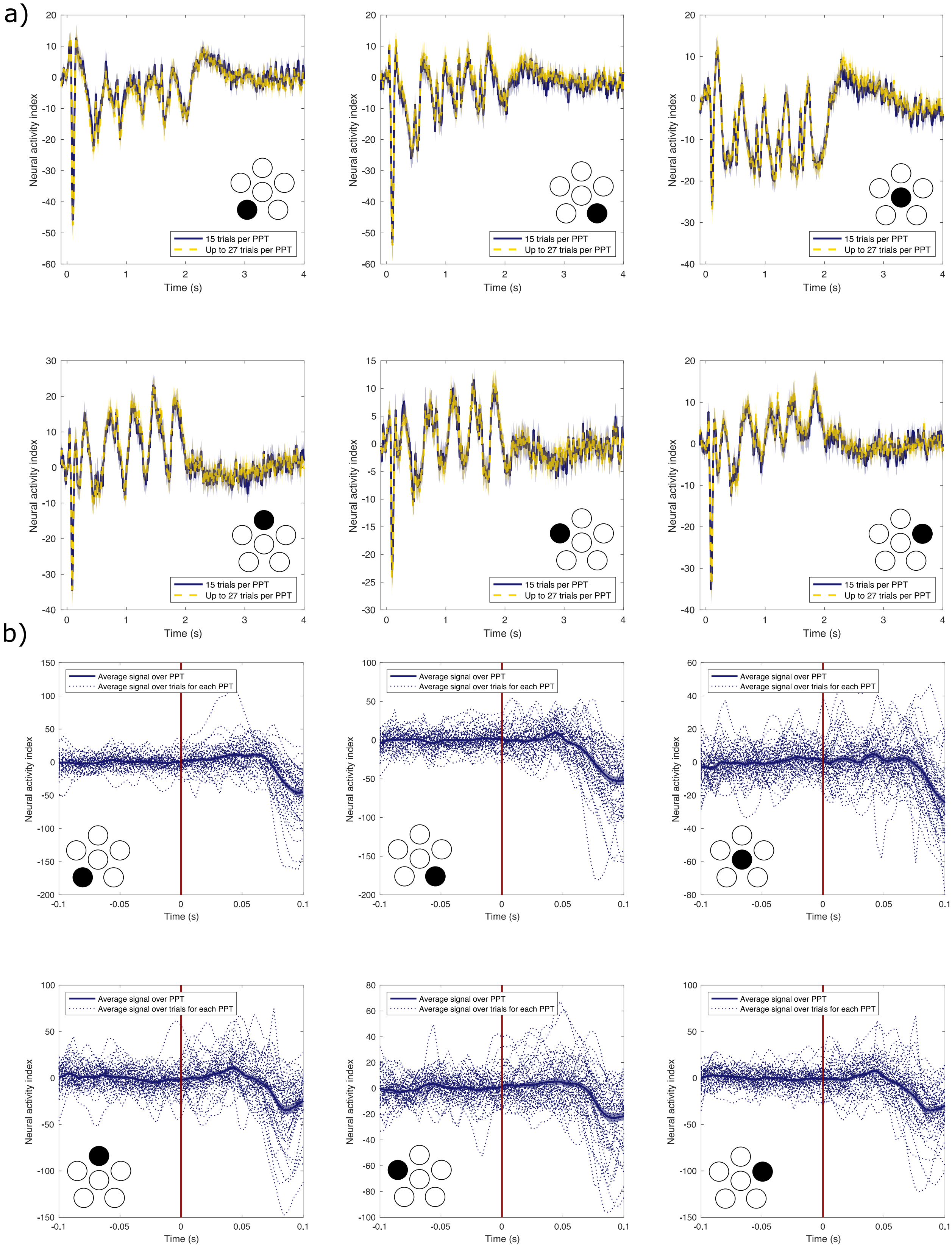

Fig. S14. Signal visualisation. a) The 6 panels represent the averaged signal for each of the ROIs, represented by the schematic diagram. The blue lines represent the averaged signal of the data used in this analysis. This is obtained by averaging the signal for each participant over their associated 15 trials. Next, we average over all participants, measuring the standard error for each participant compared to the mean. The red line contains the same analysis but using all possible trials for each participant, between 15 and 27 trials. b) Each pane represents the signal of each participant as well as the mean, of the data used in this study, focused on the baseline, prestimulus time. The red line denotes the stimulus time. The small random fluctuations before the stimulus time indicate a 'clean' baseline.

for a suitable epoch are to remove the pre-stimulus baseline of 0.1 seconds and/or to remove the final few seconds of activity where it appears the activity returns to baseline (23). In order to validate that our results hold irrespective of this choice of epoch, we consider the results of the method applied to three different epochs:

1. **Full epoch:** This is the epoch considered in the main manuscript and the SI (bar this section), which includes 4.1 seconds of activity including 0.1 seconds of pre-stimulus baseline.

2. **Baseline removed:** This epoch contains only the 4 seconds of activity post-stimulus i.e. with the baseline removed.

3. **Baseline removed / Reset removed:** This epoch contains 2.5 seconds of activity, from time 0 to 2.5 i.e. with both the baseline and the subsequent reset to baseline removed.

Figure S15 shows the 4-order irreversibilities applied to the three epoch choices. Panel a) shows that using a shorter epoch results in elevated irreversibility measurements, but that the *relative* behaviour remains the same. Furthermore, the correlations between the results for each epoch are almost perfect with $r = 1.00$ (3 s.f, ****) between Full and Baseline removed, $r = 0.927$ (3 s.f, ****) between Full and Baseline removed/Reset removed and $r = 0.926$ (3 s.f, ****) between Baseline removed and Baseline removed/Reset removed. Therefore the choice of any sensible epoch has minimal impact on the relative results.

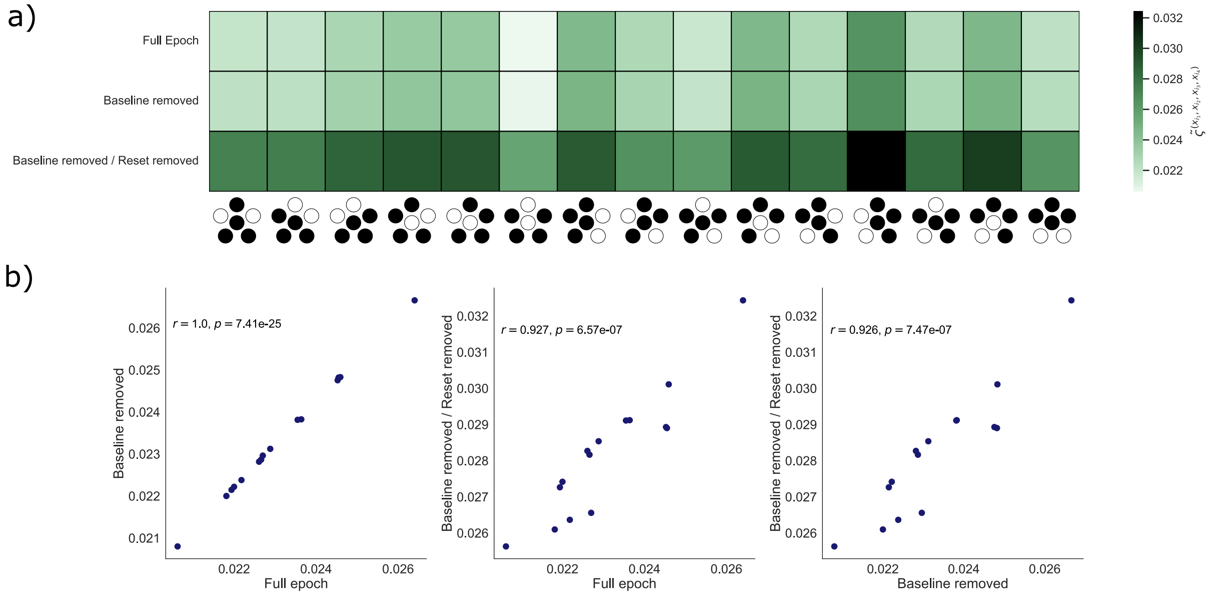

Fig. S15. Epoch comparison. a) The results of the 4-order irreversibilities for each of the three choices of epoch yield very similar results. The overall level of irreversibility is elevated for the shortest epoch. b) The correlations between the results for each epoch length are almost perfect suggesting that each choice yields equivalent results.

References

1. OJ Dunn, Multiple comparisons among means. *J. Am. Stat. Assoc.* **56**, 52–64 (1961).
2. C Battle, et al., Broken detailed balance at mesoscopic scales in active biological systems. *Science* **352**, 604–607 (2016).
3. CW Lynn, EJ Cornblath, L Papadopoulos, DS Bassett, Broken detailed balance and entropy production in the human brain. *Proc. Natl. Acad. Sci. United States Am.* **118** (2021).
4. G Lancaster, D Iatsenko, A Pidde, V Ticcinelli, A Stefanovska, Surrogate data for hypothesis testing of physical systems. *Phys. Reports* **748**, 1–60 (2018).
5. D Prichard, J Theiler, Generating surrogate data for time series with several simultaneously measured variables. *Phys. Rev. Lett.* **73** (1994).
6. CW Lynn, CM Holmes, W Bialek, DJ Schwab, Emergence of local irreversibility in complex interacting systems. *Phys. Rev. E* **106** (2022).
7. SP Strong, R Koberle, RR de Ruyter van Steveninck, W Bialek, Entropy and information in neural spike trains. *Phys. Rev. Lett.* **80** (1998).
8. SE Palmer, O Marre, MJB II, W Bialek, Predictive information in a sensory population. *Proc. Natl. Acad. Sci. United States Am.* **112**, 6908–6913 (2015).
9. E Schneidman, MJ Berry, R Segev, W Bialek, Weak pairwise correlations imply strongly correlated network states in a neural population. *Nature* **440** (2006).
10. A Treves, S Panzeri, The upward bias in measures of information derived from limited data samples. *Neural Comput.* **7**, 399—-407 (1995).
11. F Battiston, et al., The physics of higher-order interactions in complex systems. *Nat. Phys.* **17**, 1093–1098 (2021).
12. CW Lynn, CM Holmes, W Bialek, DJ Schwab, Decomposing the local arrow of time in interacting systems. *Phys. Rev. Lett.* **129** (2022).
13. C Godrèche, JM Luck, Characterising the nonequilibrium stationary states of Ornstein–Uhlenbeck processes. *J. Phys. A: Math. Theor.* **52** (2018).
14. M Gilson, E Tagliazucchi, R Cofré, Entropy production of multivariate Ornstein-Uhlenbeck processes correlates with consciousness levels in the human brain. *Phys. Rev. E* **107** (2023).
15. G Uhlenbeck, L Ornstein, On the theory of the Brownian motion. *Phys. Rev.* **36** (1930).
16. JF Donges, RV Donner, J Kurths, Testing time series irreversibility using complex network methods. *Europhys. Lett.* **102** (2013).
17. L Lacasa, B Luque, F Ballesteros, J Luque, JC Nuño, From time series to complex networks: The visibility graph. *Proc. Natl. Acad. Sci. United States Am.* **105**, 4972–4975 (2008).
18. TF Varley, O Sporns, Network analysis of time series: Novel approaches to network neuroscience. *Front. Neurosci.* **15** (2022).
19. L Lacasa, A Nuñez, E Roldán, J Parrondo, B Luque, Time series irreversibility: a visibility graph approach. *The Eur. Phys. J. B* **85** (2012).
20. L Lacasa, R Flanagan, Time reversibility from visibility graphs of nonstationary processes. *Phys. Rev. E* **92** (2015).
21. J Schnakenberg, Network theory of microscopic and macroscopic behavior of master equation systems. *Rev. Mod. Phys.* **48** (1976).
22. A Dechant, Minimum entropy production, detailed balance and Wasserstein distance for continuous-time Markov processes. *J. Phys. A: Math. Theor.* **55** (2022).
23. L Bonetti, et al., Spatiotemporal brain hierarchies of auditory memory recognition and predictive coding. *Nat. Commun.* **15** (2024).
24. L Bonetti, et al., Age-related neural changes underlying long-term recognition of musical sequences. *Commun. Biol.* **7** (2024).
25. L Bonetti, et al., Brain recognition of previously learned versus novel temporal sequences: a differential simultaneous processing. *Cereb. Cortex* **33**, 5524–5537 (2023).
26. G Fernández-Rubio, F Carlomagno, P Vuust, ML Kringelbach, L Bonetti, Associations between abstract working memory abilities and brain activity underlying long-term recognition of auditory sequences. *PNAS Nexus* **1**, 1–10 (2022).
27. G Fernández-Rubio, et al., Magnetoencephalography recordings reveal the spatiotemporal dynamics of recognition memory for complex versus simple auditory sequences. *Commun. Biol.* **5** (2022).
28. G Fernández-Rubio, et al., Investigating the impact of age on auditory short-term, long-term, and working memory. *Psychol. Music.* **52**, 187–198 (2024).
29. DMP Jayakody, PL Friedland, RN Martins, HR Sohrabi, Impact of aging on the auditory system and related cognitive functions: A narrative review. *Front. Neurosci.* **12** (2018).
30. S Taulu, J Simola, Spatiotemporal signal space separation method for rejecting nearby interference in meg measurements. *Phys. Medicine Biol.* **51**, 1–10 (2010).
31. M Woolrich, L Hunt, A Groves, G Barnes, MEG beamforming using Bayesian PCA for adaptive data covariance matrix regularization. *Neuroimage* **57**, 1466–1479 (2011).
32. R Oostenveld, P Fries, E Maris, JM Schoffelen, Fieldtrip: Open source software for advanced analysis of MEG, EEG, and invasive electrophysiological data. *Comput. Intell. Neurosci.* (2011).
33. MW Woolrich, et al., Bayesian analysis of neuroimaging data in FSL. *Neuroimage* **45**, S173–S186 (2009).

34. K Friston, J Ashburner, S Kiebel, T Nichols, W Penny, *Statistical Parametric Mapping: The Analysis of Functional Brain Images.* (Academic Press), (2006).
35. D Mantini, et al., A signal-processing pipeline for magnetoencephalography resting-state networks. *Brain Connect.* **1**, 49–59 (2011).
36. A Hillebrand, GR Barnes, Beamformer analysis of MEG data. *Int. Rev. Neurobiol.* **68**, 149–171 (2005).
37. MX Huang, JC Mosher, RM Leahy, A sensor-weighted overlapping-sphere head model and exhaustive head model comparison for MEG. *Phys. Medicine Biol.* **44**, 423–440 (1999).
38. MJ Brookes, et al., Beamformer reconstruction of correlated sources using a modified source model. *Neuroimage* **34**, 1454–1465 (2007).
39. G Nolte, The magnetic lead field theorem in the quasi-static approximation and its use for magnetoencephalography forward calculation in realistic volume conductors. *Phys. Medicine Biol.* **48**, 3637–3652 (2003).
40. MX Huang, et al., Commonalities and differences among vectorized beamformers in electromagnetic source imaging. *Brain Topogr.* **16**, 139–158 (2004).
41. HT Luckhoo, MJ Brookes, MW Woolrich, Multi-session statistics on beamformed MEG data. *Neuroimage* **95**, 330–335 (2014).
42. L Parkkonen, Instrumentation and data preprocessing in *MEG: An introduction to methods.* (Oxford University Press), (2010).
